# Beyond the Gini index: a poverty-normalized spatial Bayesian model for assessing territorial inequality

**DOI:** 10.1186/s12939-026-02890-3

**Published:** 2026-05-26

**Authors:** Xavier Perafita, Marta Solans, Laura Vilà-Quintana, Maria Antònia Barceló, Marc Saez

**Affiliations:** 1 Observatori — Organisme Autònom de Salut Pública de la Diputació de Girona (Dipsalut), Girona, 17003 Spain; 2 Research Group on Statistics, Econometrics and Health (GRECS), University of Girona, Girona, 17003 Spain; 3 Centro de Investigación Biomédica en Red de Epidemiología y Salud Pública, Instituto de Salud Carlos III, Madrid, 28029 Spain; 4 Digestive Diseases and Microbiota Group, Girona Biomedical Research Institute (IDIBGI), Salt, 17190 Spain

**Keywords:** Income inequality, Gini index, Poverty rate, Bayesian spatial modelling, Spatial econometrics

## Abstract

**Background:**

The Gini index is one of the most widely used measures of income inequality. However, it has limitations in allowing reliable comparisons across areas with different socio-economic structures, particularly in small populations, where the index can be highly unstable. This study explores a simple modification of the Gini index that can overcome some of these limitations.

**Methods:**

We propose a modification of the original Gini index that incorporates the poverty rate as an offset. Combined with Bayesian spatial modelling, this approach improves the precision and comparability of small-area estimates, by providing measures of inequality conditional on local poverty levels. To illustrate it, income data from 8,043 Spanish municipalities for the year 2022 were used, and the spatial patterns of the normalized Gini index were compared with those obtained from the original index.

**Results:**

The normalized Gini index revealed spatial inequality patterns that were overlooked by the original index. The new approach identified municipalities with higher levels of inequality than expected given their poverty rates in Andalusia, Extremadura, and Murcia, in contrast to lower inequality in Catalonia, Basque Country, Madrid, and Balearic Islands. In addition, while the original Gini index increased with population density and urbanization, the normalized version showed an opposite trend, with higher relative inequality observed in rural and low-density municipalities after accounting for local poverty levels.

**Conclusions:**

Given its simplicity, reliance on widely available indicators, and adaptability to different territorial scales, the proposed method offers a useful measure for describing income inequality conditional on a region’s poverty level and informing local redistributive policies.

**Supplementary Information:**

The online version contains supplementary material available at 10.1186/s12939-026-02890-3.

## Introduction

Income inequality has been a central concern in social sciences since the early 20th century, although its historical roots run much deeper. This has led to the development of over 50 inequality indices [1], with the Gini index [2] being, by far, the most widely adopted measure. The Gini index is based on the Lorenz curve [3], which graphically represents the percentage of total income earned by the cumulative percentage of the population (Figure S1). The Gini index can be calculated as the ratio of the area between the perfect equality line (45º) and the Lorenz curve (A) divided by the total area under the perfect equality line (A + B). Thus, the Gini coefficient summarises the entire distribution into a single value, ranging from 0 (perfect equality) to 1 (maximum inequality, wherein all income is earned by one individual).

Despite its widespread use in economic analysis and policymaking due to its ease of construction and interpretation, the Gini index has several well-documented limitations. First, it does not distinguish how wealth is distributed within the population [4], making it difficult to distinguish whether inequality is driven by a wealthy elite or by a large portion of the population living in poverty. Second, it is highly sensitive to changes in the middle of the income distribution and less sensitive to transfers among the very rich and very poor, limiting its ability to capture the impact of redistributive policies [5]. Third, when comparing different territories, it does not clearly indicate which region has higher inequality when Lorenz curves intersect [5]. Finally, it does not account for differences in the cost of living, the informal economy, or tax systems, which can distort cross-territory inequality comparisons [6].

Estimating the Gini index at small geographical scale presents further challenges - data instability, the presence of extreme values [6, 7] and small sample bias [6, 8], can lead to less reliable estimates. To address this, researchers typically rely on parametric estimations of the Lorenz curve, but there is no single distribution that can accurately capture income distribution. While early studies used simple parametric approaches [9–11], later models introduced multi-parameter specifications to improve accuracy [6, 7, 12]. More recently, Bayesian techniques, machine learning methods, and alternative distributions have been integrated to enhance estimation accuracy [6, 7, 13].

Previous research on how to overcome part of the Gini’s limitations follows two main approaches. The first proposes replacing the Gini coefficient with other inequality single-measures [14] however, none of them is able to completely describe the entire income distribution [15] and, although some practical frameworks have been proposed [16], there is a lack of consensus on which may be the best choice in each situation. The second approach involves adapting the Gini coefficient, generally by combining it with another measure, such as the income share held by the top and bottom 10% [12, 17–19] or the relative median deviation [20, 21] in order to capture additional features of the income distribution.

In parallel, the literature on spatial inequality has highlighted that inequity measures can be strongly influenced by spatial dependence and the scale of analysis. Several studies have pointed out that spatial autocorrelation and territorial clustering can substantially alter the interpretation of inequity indicators, especially when working with aggregated geographical units [22–25]. These studies typically focus on regional or NUTS-2/NUTS-3 scales, but analyses at more disaggregated scales, such as the municipal (LAU-2) level analysis, pose additional challenges related to the instability of estimates and the presence of complex spatial patterns.

To address these gaps, we propose a simple modification of the Gini index that incorporates the poverty rate as an offset. The poverty rate, defined as the proportion of population below a defined poverty threshold (in our context, the 60% below the median of the national income distribution), is a Sustainable Development Goal (SDG) indicator that is widely available across countries [26]. This normalization does not aim to measure poverty itself, but rather to estimate inequality conditional on the level of poverty. By incorporating the poverty rate as an offset, the model defines an expected level of inequality for each municipality and captures deviations from this expected pattern. The normalized Gini index therefore indicates whether observed inequality is higher or lower than would be expected given the local poverty rate. In addition, we use a Bayesian approach to estimate both the original and this normalized Gini index, which improves the precision of small areas estimates and reduces instability related to small population sizes.

To illustrate this approach, we have estimated both the original Gini index and its adapted version across Spain’s municipalities. Although Spain ranks 15th globally in terms of Gross Domestic Product (GDP), it falls to 46th place in GDP per capita, highlighting substantial imbalances in resource distribution, partly captured by a Gini index of 31.2 in 2024 [27]. In this context, this adaptation helps identify municipalities where inequality is higher or lower than we would expect given their poverty level. It also makes it possible to distinguish municipalities with similar poverty levels but different internal income distributions. This adjustment minimizes distortions that arise when comparing the original index across small territories with significantly different socioeconomic conditions, offering a more intuitive and policy-relevant tool for inequality assessment.

## Methods

### The Gini index normalization

The Gini coefficient (ranging from 0 to 1) was modelled through a Beta distribution:

1 $$\:B\left(a,\beta\:\right)={\int\:}_{0}^{1}{t}^{\alpha\:-1}{\left(1-t\right)}^{\beta\:-1}dt$$

In particular, this Beta distribution depends on two parameters, the expected mean of the Gini index for each municipality ($\:{\mu\:}_{i})$ and the precision ($\:\phi\:$):

2 $$Gin{i_i} \sim Beta\left( {{\mu _i}\cdot\phi ,\left( {1 - {\mu _i}} \right)\cdot\phi } \right)$$

To normalise the Gini index, we specified a generalised linear mixed model (GLMM) for the Gini variable:

3 $$\begin{aligned} logit\left( {u_i^{Gini}} \right) = & {\beta _0} + {\eta _i} + S\left( {municipalit{y_i}} \right) + \\ & offset\left( {\log \left( {poverty\_rat{e_i}} \right)} \right) \\ \end{aligned} $$

where $\:{u}_{i}^{Gini}$ denotes the expected value of the Gini index for municipality $\:i$, i.e., $\:{u}_{i}^{Gini}={\mu\:}_{i}$.

The normalized Gini index corresponds to the posterior mean of $\:{u}_{i}^{Gini}$, which was modelled on its original 0–1 scale and is presented in all reported results and figures on a 0–100 scale (i.e., Gini × 100) for ease of interpretation, so that differences can be interpreted as Gini points.

The percentage of population below the 60% of the national median income (*poverty_rate*) was included in the model as an offset, to account for its structural role in shaping observed inequality. By specifying the poverty rate as an offset, a coefficient equal to one is imposed, so that poverty is treated as a fixed structural reference rather than as an estimated covariate. Under this specification, the model captures deviations in observed inequality relative to what would be expected given the local poverty rate. To capture unobserved confounders and improve model fit, two random effects were included: an unstructured random effect $\:{(\eta\:}_{i})$ and a spatially structured random effect $\:{(S}_{i})$ The unstructured effect $\:{(\eta\:}_{i})$ was specified as independent and identically distributed (iid) applied at the municipal level to account for unobserved factors, whereas the spatially structured effect ($\:{S}_{i}$) accounts for spatial dependence, given that geographically near areas tend to exhibit more similar outcomes. The structured effect was modelled using a Matérn covariance function, allowing for flexible characterisation of spatial autocorrelation. The covariance between two spatial locations and is defined as:

4 $$\begin{aligned} (Cov\left( {S\left( {{x_i}} \right),S\left( {{x_{i'}}} \right)} \right) = & \frac{{{\sigma ^2}}}{{{2^{\nu - 1}}\Gamma \left( \nu \right)}}{\left( {\kappa \parallel {x_i} - {x_{i'}}\parallel } \right)^\nu } \\ & {{\mathrm{K}}_\nu }\left( {\kappa \parallel {x_i} - {x_{i'}}\parallel } \right) \\ \end{aligned} $$

Inference was conducted from a Bayesian perspective using the integrated nested Laplace approximation (INLA) [28, 29]. Random effects were defined using a multivariate Gaussian distribution with mean 0 and precision matrix kΣ, where k is a scaling constant and Σ defines the spatial correlation structure. We employed penalised complexity (PC) priors to ensure robustness [30].

### Alternative specifications of the poverty rate

We explored three alternative strategies for incorporating the poverty rate into the model: (i) as an offset, as previously detailed, (ii) as a covariate, or (iii) as weighting factors. Model performance were evaluated using the Deviance Information Criterion (DIC) [31], the Watanabe–Akaike Information Criterion (WAIC) [32], and the logarithm of the pseudo marginal likelihood (LPML) [33], and posterior uncertainty was assessed through the average width of 95% credibility intervals (CI) (Tables S1 and S2). Additionally, we examined the distribution of the predicted values, particularly their range and standard deviation, to assess whether each specification preserved meaningful variability across municipalities. Model selection was based on the best balance between goodness-of-fit and the preservation of variability in the predicted values.

Although the covariate model achieved the lowest DIC, WAIC, and LPML values, its predictive range was extremely narrow (0.274–0.311) suggesting an excessive smoothing that may attenuate the influence of poverty across municipalities. Conversely, the weighted model showed an even more restricted distribution, producing almost constant predictions and narrow credibility intervals. In contrast, the offset model showed a broader predictive range (0.061–0.515), closer to the observed variability of the Gini index (0.203–0.442).

We further assessed the explanatory power by computing Pearson correlation coefficients between each modelled index and five socioeconomic indicators: poverty rate, extreme poverty rate, 80/20 income ratio, per capita income, and the higher-to-lower education ratio (Table S3). Although the offset and covariate models exhibited nearly identical correlations with these indicators, their interpretability differed markedly. The covariate model displayed oversmoothing, limiting its ability to reflect territorial variability, while the weighted model underestimated variability, producing almost constant predictions. In contrast, the offset model maintained a predictive range closer to the observed Gini values, offering a more robust and interpretable representation of income distribution patterns.

Overall, the comparison of the three modelling approaches evidenced that incorporating the poverty rate as an offset provided the best balance between interpretability and the ability to capture the territorial variability. Therefore, this was the approach selected to normalize the Gini index with the poverty rate in our study.

### Study design and data availability

To illustrate the use of this normalized Gini coefficient, a cross-sectional ecological study covering 8,043 Spanish municipalities (all of them excluding Canary Islands) was conducted. Municipal-level data for 2022 was obtained from the Household Income Distribution Atlas (HIDA), which is openly provided by Spanish National Statistics Institute [34].

The Gini index and the poverty rate were available for all the municipalities, except those with less than 100 income tax declarants - for confidentiality reasons - or those with extreme values. To ensure territorial continuity in our analysis and prevent the loss of municipalities with partial data, especially those with smaller populations, those values were imputed. The full dataset, including Canary Islands (8,138 municipalities), was used in the imputation procedure. Particularly, missing data affected 1,366 municipalities for the Gini index and 4,005 for the poverty rate (Table S4).

The imputation was based on a set of socioeconomic and demographic predictors available at the municipal level, based on their theoretical and empirical association with income distribution and poverty. Before imputation, we applied a preliminary data filtering process to reduce multicollinearity and avoid redundancy among predictors. Variables with pairwise correlation |r| ≥ 0.8 were considered highly collinear and excluded from the imputation model [23]. The final included variables where: net income per capita, net household income, main sources of income (pensions, unemployment benefits, other social benefits, and other income), the 80/20 income ratio (P80/P20), population below 60% and 200% of the median income, mean age, proportion of single-person households, total population size, proportion of Spanish nationals, and educational attainment (primary, lower secondary, and higher education). Some variables closely related to the poverty rate (e.g., population below 60% of the median income) were included as auxiliary predictors to improve imputation accuracy, following standard recommendations in multiple imputation practice.

Subsequently, several imputation methods were evaluated, among which the predictive mean matching (PMM) [35] was selected because it better preserved the observed distribution of the poverty rate (Figure S2). Briefly, the PMM imputes a missing value by:

5 $$\:{x}_{ij}^{miss}\sim PMM({\widehat{x}}_{ij,}\left\{{x}_{mj}^{obs}\right\}m\in\:{N}_{k})$$

where $\:{x}_{ij}^{miss}$ represents the missing observation $\:i$ for variable $\:j$; $\:{\widehat{x}}_{ij}$ is the predicted value from a regression model based on the other observed variables, $\:\left\{{x}_{mj}^{obs}\right\}$ refers to the set of fully observed values for variable i; and $\:\left\{{x}_{mj}^{obs}\right\}m\in\:{N}_{k}$ denotes the 5 nearest observed values to the predicted value $\:{\widehat{x}}_{ij}$. This imputation procedure does not explicitly incorporate geographic proximity, although spatial patterns may be indirectly reflected through correlated predictor variables.

A total of 25 imputed datasets were generated. All subsequent analyses were conducted separately within each imputed dataset, and the resulting estimates were combined using Rubin’s rules [36]. Specifically, pooled point estimates were calculated as the mean across imputations, and total variance was obtained by combining within- and between-imputation variability. The final PMM model was then assessed through density plots, quantile–quantile plots, and comparisons of summary statistics, skewness, and kurtosis between observed and imputed values (Figures S3–S4; Table S5). Although the Kolmogorov–Smirnov test indicated a statistically significant difference, the magnitude of this difference was small (D = 0.049), suggesting good agreement between the observed and imputed distributions.

To assess the impact of the imputation on the results, a sensitivity analysis was conducted by restricting the analysis to municipalities with complete observed data for both the Gini index and the poverty rate. The main Bayesian model was re-estimated using this complete-case dataset, and the resulting estimates were compared with those obtained from the imputed-data analysis. This approach allowed us to evaluate whether the inclusion of imputed values influenced the main findings (Table S6).

Overall, the Gini index and its normalized version were computed by municipality, and by several regional divisions: NUTS-1 (large socioeconomic territories), NUTS-2 (autonomous communities), NUTS-3 (provinces). In addition, and to explore potential structural differences, municipalities were stratified by (i) population size (< 5,000; 5,001 to 10,000; 10,001 to 20,000; 20,001 to 30,000; 30,001 to 50,000; 50,001 to 100,000; 100,001 to 500,000; and > 500,000 inhabitants), (ii) level of urbanisation, following Eurostat degree of urbanisation (DEGURBA) criteria [37] (i.e., urban: density > 1,500 inhabitants/km² and ≥ 50,000 inhabitants; semi-urban: 300 to 1,500 inhabitants/km² and ≥ 5,000 inhabitants; and rural: density < 300 inhabitants/km² or not meeting the other criteria), and (iii) capital status (i.e. capitals and non-capitals).

The explanatory power of the original and normalized Gini indices was explored through Pearson correlation coefficients between each index and five socioeconomic indicators: poverty rate, extreme poverty rate, 80/20 income ratio, per capita income, and the higher-to-lower education ratio.

All calculations and visualisations were performed using R version 4.1.3, with the following libraries: mice (v3.14.0), sf (v4.1.3), INLA (v25.2.10), leaflet (v3.3.3), htmltools (v0.5.8.1), boot (v1.3-31), and plotly (v4.10.1).

## Results

**Fig. 1:**
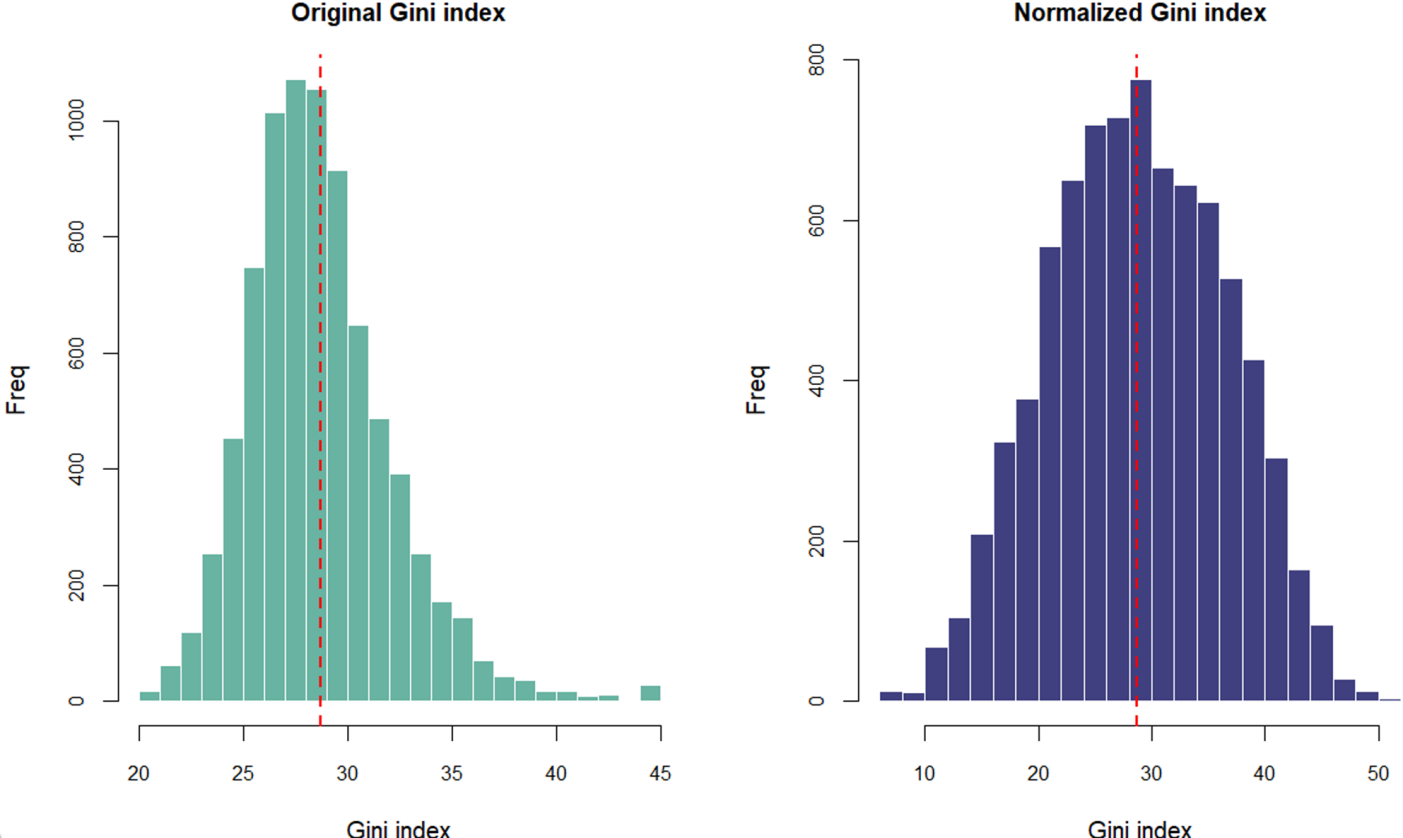
Distribution of **A**) the original Gini index and **B**) the normalized Gini index in Spain (2022)

The original Gini index and its normalized version were computed for 8,043 municipalities in Spain for 2022. Figure 1 provides an overview of their distribution. The original Gini index appeared more peaked and apparent right-censoring, with a mean of 28.7, a median of 28.1, and a relatively narrow spread (SD = 3.4, min-max = 20.3–44.2). In contrast, the normalized version presented greater dispersion (SD = 7.8, min-max = 6.1–51.5) and fewer outliers, while maintaining the same mean and median of 28.7. The similar mean and median suggest that the normalization did not materially shift the central tendency of the index, but instead increased its dispersion across municipalities, revealing territorial heterogeneity that was less apparent in the original distribution.

Normalization increased the dispersion of the Gini index within autonomous communities (NUTS-2) (Figure S5, Table 1). This accentuated the contrast between regions with higher inequality than expected given local poverty levels, such as Andalusia (mean = 36.5; SD = 5.91; range = 11.4–50.6), Extremadura (mean = 37.1; SD = 5.12; range = 17.9–49.7) or Castilla-La Mancha (mean = 31.4; SD = 6.5; range = 10.9–49.7), and wealthier regions, such as the Basque Country (mean = 21.0, SD = 7.3, range = 6.1–45.7), the Balearic Islands (mean = 22.8, SD = 4.5, range = 13.5–31.4), Catalonia (mean = 23.2, SD = 7.1, range = 6.1–49.9), or the Community of Madrid (mean = 26.2, SD = 6.2, range = 10.5–41.8). Results at a province-level (NUTS-3) are provided in Table S7, with notable examples such as Guipuzkoa, Eivissa/Formentera, Cáceres or Jaén, where the mean difference between both indexes reached almost 10 points.

**Table 1:** Summary statistics of the original Gini index and the normalized Gini index at NUTS-1 and NUTS-2 level in Spain (2022)

Zone	*N*	Original Gini index			Normalized Gini index		
		Mean (SD)	Median (Q1-Q3)	Min-Max	Mean (SD)	Median (Q1-Q3)	Min-Max
**Country**							
Spain	8043	28.71 (3.4)	28.3 (26.4–30.5)	20.3–44.2	28.7 (7.8)	28.6 (23.0-34.5)	6.1–51.5
**NUTS 1** ^**1**^							
Northwest	493	27.2 (2.4)	26.9 (25.6–28.5)	21.4–40.7	28.4 (6.0)	28.0 (23.9–32.0)	10.8–51.5
Northeast	1428	29.2 (3.8)	28.6 (26.6–31.1)	20.3–44.2	26.2 (7.5)	26.0 (20.7–31.6)	6.1–51.5
Community of Madrid	179	30.7 (3.4)	30.4 (28.3–32.8)	23.6–42.6	26.2 (6.2)	26.4 (21.6–30.2)	10.5–41.8
Centre	3555	28.6 (3.5)	28.2 (26.3–30.4)	20.3–44.2	29.6 (7.2)	29.4 (24.4–34.8)	10.1–49.7
East	1556	28.9 (3.3)	28.5 (26.6–30.7)	20.5–44.2	25.2 (7.5)	24.9 (19.8–30.3)	6.1–49.9
South	832	28.7 (3.1)	28.4 (26.5–30.3)	21.4–44.2	36.4 (5.8)	37.1 (33.1–40.3)	11.4–50.7
**NUTS 2** ^**2**^							
Galicia	313	26.9 (2.3)	26.8 (25.4–28.3)	21.4–40.7	27.2 (5.4)	26.9 (23.5–30.4)	12.5–51.5
Principality of Asturias	78	27.8 (2.4)	27.6 (26.1–29.4)	21.8–36.3	28.6 (5.0)	28.1 (25.5–31.9)	17.2–45.7
Cantabria	102	27.4 (2.3)	27.2 (25.9–28.6)	21.7–33.6	31.9 (7.2)	33.2 (25.6–38.0)	10.8–43.5
Basque Country	251	28.6 (3.3)	28.3 (26.4–30.3)	21.4–44.2	21.0 (7.4)	19.5 (16.2–24.8)	6.1–45.7
Navarra	272	30.9 (4.9)	30.3 (27.1–34.0)	20.4–44.2	27.1 (6.6)	27.1 (22.3–31.5)	11.7–51.5
La Rioja	174	29.9 (3.3)	29.5 (27.6–31.7)	22.9–44.2	29.9 (6.7)	30.0 (24.9–34.8)	14.8–45.1
Aragon	731	28.5 (3.3)	28.2 (26.4–30.4)	20.3–42.5	26.8 (7.2)	26.6 (21.6–32.0)	6.3–47.3
Community of Madrid	179	30.7 (3.4)	30.4 (28.3–32.8)	23.6–42.6	26.2 (6.2)	26.4 (21.6–30.2)	10.5–41.8
Castile-Leon	2248	28.5 (3.5)	28.2 (26.2–30.4)	20.3–44.2	27.5 (6.7)	27.2 (22.8–32.4)	10.1–47.8
Castile-La Mancha	919	29.5 (3.3)	29.0 (27.3–31.1)	20.3–44.2	31.4 (6.5)	31.7 (27.1–36.0)	10.9–49.7
Extremadura	388	26.7 (2.7)	26.6 (25.0-28.2)	20.3–37.7	37.1 (5.1)	37.4 (33.9–40.9)	17.9–49.7
Catalonia	947	28.9 (3.4)	28.5 (26.6–30.8)	20.5–44.2	23.3 (7.1)	22.9 (18.1–28.1)	6.1–49.9
Valencian Community	542	28.6 (3.1)	28.2 (26.4–30.3)	20.6–38.4	28.9 (7.1)	29.2 (23.7–34.5)	12.3–45.7
Balearic Islands	67	30.4 (2.8)	30.0 (28.7–31.9)	23.1–40.6	22.8 (4.5)	23.4 (20.3–26.4)	13.5–31.4
Andalusia	785	28.6 (3.1)	28.3 (26.5–30.3)	21.4–44.2	36.5 (5.9)	37.2 (33.1–40.5)	11.4–50.7
Region of Murcia	45	28.8 (2.0)	28.8 (27.5–29.8)	24.2–33.1	35.2 (3.2)	34.9 (32.7–37.4)	28.1–41.7

N, count; Q1, quartile 1; Q3, quartile 3; SD, standard deviation

^1^List of NUTS-2 regions included in each category: Northwest (Galicia, the Principality of Asturias, and Cantabria), Community of Madrid (Community of Madrid), Centre (Castile-Leon, Castile-La Mancha and Extremadura), East (Catalonia, Valencian Community and Balearic Islands), and South (Andalusia and Region of Murcia)

^2^Ceuta and Melilla are excluded from the analysis, as they are designated autonomous cities rather than autonomous communities

**Fig. 2:**
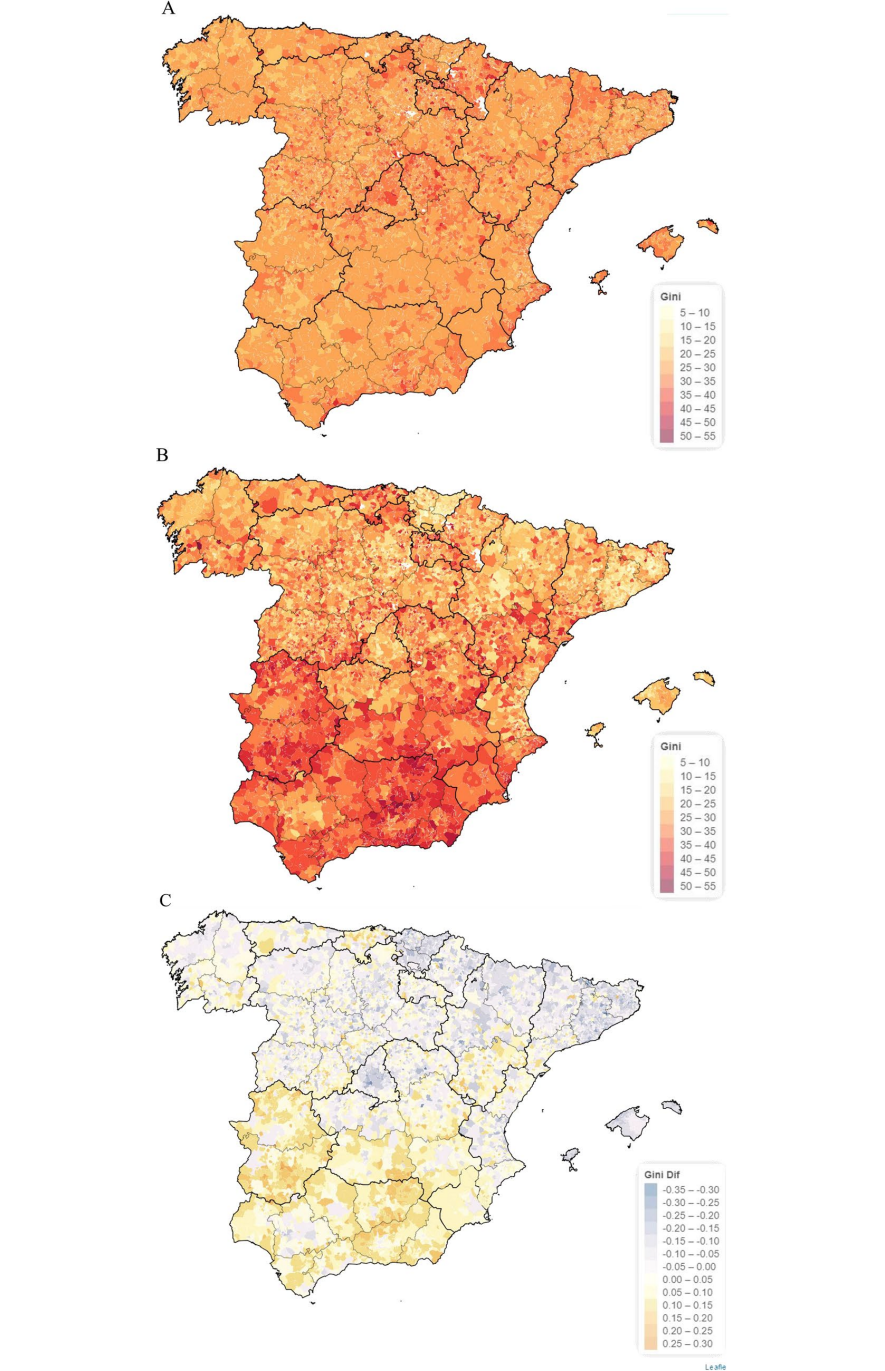
Geographical representation at a municipality level in Spain (2022) of the **A**. original Gini index. **B**. the normalized Gini index, and **C** the difference between original Gini index andnormalized Gini index

The geographical representation of both indices at a municipality level is shown in Fig. 2, and is further detailed for each NUTS-1 in Figure S6. Broadly, the distribution of the original index remained relatively homogeneous, with variations between municipalities but no evident spatial pattern (Fig. 2a). In contrast, the normalized version (Fig. 2b) displayed a wider range of values and evidenced higher indices in the south (i.e. Andalusia and Extremadura) and lower indices in the northeast (i.e. Basque Country and Catalonia), the Community of Madrid, and Balearic Islands. The difference between both indices is displayed in Fig. 2c, which evidenced that most of these changes occurred in regional capitals and their surrounding areas. As sensitivity analyses, other socio-economic indicators by municipality were mapped in Figure S7. In line with the spatial pattern observed with the normalized Gini index, extreme poverty (Figure S7a) was mostly confined to south regions, while rates were markedly lower in the north and north-east area. Likewise, higher rates of individuals with higher education (Figure S7b) and or gross net income per person (Figure S7c) were mostly observed in the north, northeast and metropolitan area of Madrid, Barcelona, and Basque Country, in contrast to lower rates observed in the south, east, and centre of Spain.

**Fig. 3:**
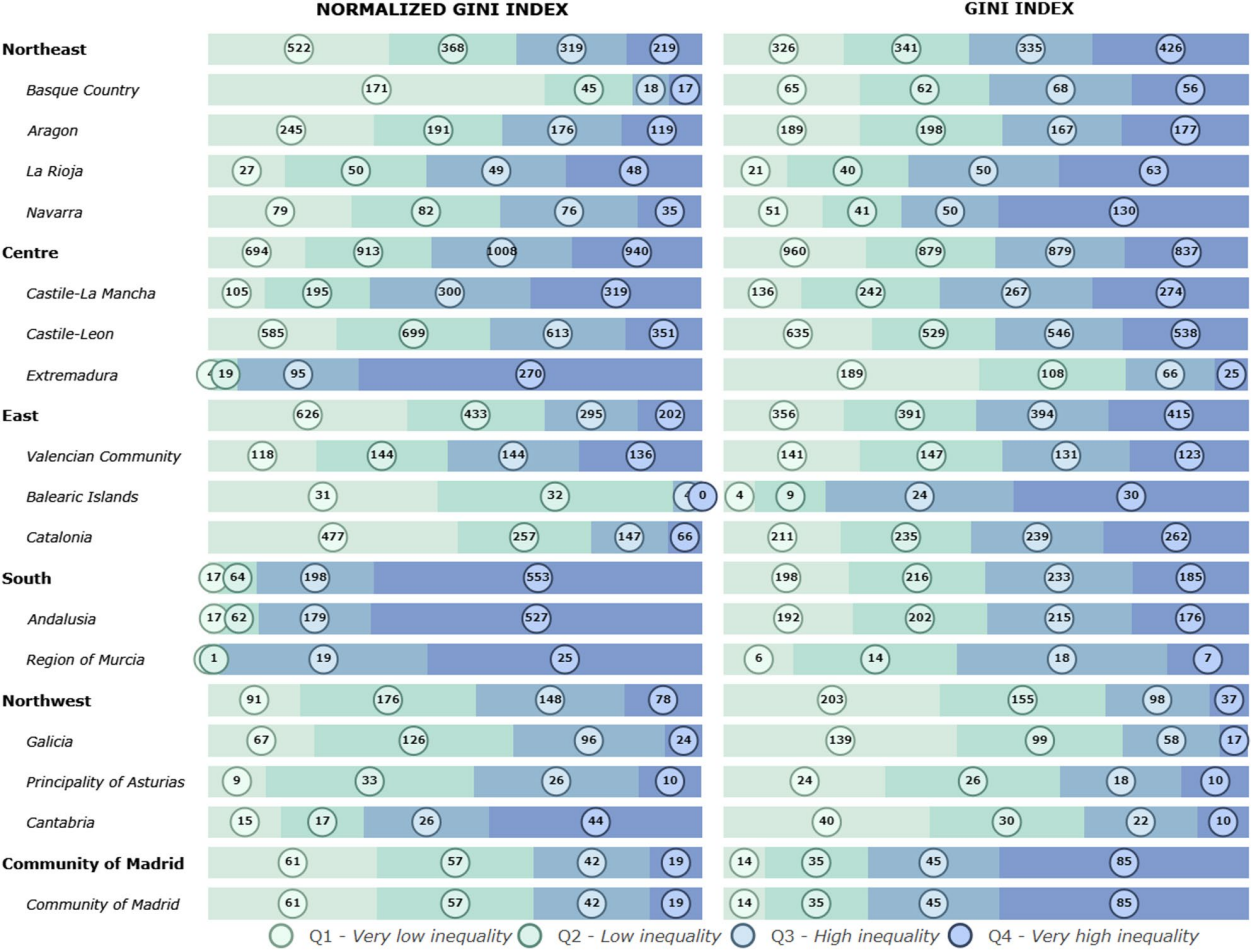
Distribution of municipalities across inequality quartiles at NUTS-1 and NUTS-2 in Spain (2022), based on the normalized (left) and the original (right) Gini index. The bubbles indicate the number of municipalities belonging to each quartile of inequality

Figure 3 shows the distribution of municipalities by index quartile, where Q1 represents very low inequality and Q4 very high inequality. In Extremadura, the number of municipalities with high inequality (Q3 and Q4) rose from 91 (23%) to 365 (94%), in line with Andalusia (50% to 90%) and the Region of Murcia (55% to 98%). An opposite pattern was observed in the Balearic Islands (81% to 6%), the Basque Country (49% to 14%), the Community of Madrid (73% to 34%), and Catalonia (from 53% to 23%).

Table 2 presents a comparison of both indices across Spanish municipalities, grouped by population size, level of urbanization, and capital status. The original Gini index increased with population size, peaking in cities with over 500,000 inhabitants (mean = 33.9; SD = 2.65; range = 29.6–37.7). However, after normalization, this pattern was partially reversed, and large municipalities showed the lowest inequality levels relative to their poverty rate (mean = 24.11; SD = 5.81; range = 14.76–32.82). Notably, the smallest municipalities (< 5,000 inhabitants) displayed the widest range of normalized values (min-max = 6.13–51.5), reflecting the presence of both the lowest and highest inequality indices across Spain. A clearer trend was observed with the level of urbanization: before normalization, the Gini index increased with the level of urbanization. After normalization, the trend inverted, with lower indices in urban areas (mean = 26.14; SD = 6.43; range = 13.82–42.12), followed by semi-urban areas (mean = 26.43; SD = 8.41; range = 9.43–45.46), and the highest values concentrated in rural areas (mean = 28.84; SD = 7.72; range = 6.13–51.5), indicating that inequality remained higher than expected given local poverty levels in these territories. A similar shift was observed when comparing capitals and non-capitals; although capitals had higher original Gini values (mean = 31.76, SD = 2.02, range = 27.7–37.7), they displayed lower levels of inequality relative to what would be expected given their poverty level (mean = 26.71 vs. 28.7).

**Table 2:** Summary statistics of the original Gini index and the normalized Gini index by size population, urban degree and capital status in Spain (2022)

Zone	*N*	Gini index			Normalized Gini index		
		Mean (SD)	Median (Q1-Q3)	Min-Max	Mean (SD)	Median (Q1-Q3)	Min-Max
**Population size**							
Less than 5,000	6,797	28.7 (3.5)	28.2 (26.3–30.5)	20.3–44.2	28.8 (7.8)	28.7 (23.2–34.5)	6.1–51.5
5,001 to 10,000	526	28.2 (2.6)	27.9 (26.3–29.5)	21.4–44.2	27.9 (8.2)	27.8 (21.6–34.6)	9.4–48.8
10,001 to 20,000	336	28.8 (2.5)	28.5 (27.2–30.2)	22.4–37.5	27.7 (7.6)	28.0 (21.5–33.9)	10.2–44.1
20,001 to 30,000	142	29.6 (2.4)	29.3 (27.7–30.8)	24.3–38.1	29.2 (7.5)	29.6 (23.2–35.4)	12.8–43.9
30,001 to 50,000	100	29.6 (2.4)	29.4 (28.1–30.5)	25.2–39.6	28.9 (7.1)	29.3 (24.4–34.2)	13.5–46.1
50,001 to 100,000	82	31.3 (3.0)	30.8 (29.2–32.6)	26.5–40.6	27.3 (8.2)	26.1 (21.4–33.7)	10.5–45.5
100,001 to 500,000	54	31.5 (2.6)	31.2 (30.0-32.4)	26.3–42.6	28.5 (5.3)	27.7 (24.4–32.1)	18.2–39.5
Over 500,000	6	33.9 (2.7)	33.9 (33.3–34.9)	29.6–37.7	24.1 (5.8)	24.3 (23.0-25.5)	14.7–32.8
**Level of urbanization** ^1^							
Urban	70	32.0 (3.6)	31.4 (29.3–34.2)	26.3–42.6	26.1 (6.4)	25.3 (22.5–28.7)	13.8–42.1
Semi-urban	414	29.4 (2.9)	29.3 (27.5–31.3)	23.0-39.6	26.4 (8.4)	25.8 (19.7–32.9)	9.4–45.5
Rural	7,559	28.6 (3.4)	28.2 (26.3–30.4)	20.3–44.2	28.8 (7.7)	28.7 (23.3–34.6)	6.1–51.5
**Capital** ^**2**^							
Capital	48	31.8 (2.0)	31.4 (30.5–32.8)	27.7–37.7	26.7 (5.5)	25.9 (23.7–30.9)	14.8–38.2
Non-capital	7,995	28.7 (3.4)	28.3 (26.4–30.5)	20.3–44.2	28.7 (7.8)	28.6 (23.0-34.5)	6.1–51.5

N, count; Q1, quartile 1; Q3, quartile 3; SD, standard deviation

^1^Urban municipalities are defined as those with a density > 1,500 inhabitants/km² and a population ≥ 50,000. Semi-urban municipalities are defined as those with a density between 300 and 1,500 inhabitants/km² and a population ≥ 5,000. Rural municipalities are defined as those with a density < 300 inhabitants/km² or not meeting the other criteria

^2^Capital municipalities are defined as those that serve as the administrative capital of a province

The explanatory capacity of the two indices is presented in Table 3. Both Pearson correlation coefficients and regression coefficients were statistically significant in all the models. Regarding correlations, the normalized Gini index was more correlated with extreme poverty rate (*r* = 0.664), per capita income (*r* = − 0.569), and the ratio of higher-to-lower educational levels (*r* = − 0.390), compared with the classical Gini index (*r* = 0.289, *r* = 0.199, and *r* = 0.156, respectively). Conversely, the correlation with the 80/20 income ratio was higher for the original Gini index (*r* = 0.676) than for the normalized version (*r* = 0.130). Regarding regression coefficients (β), these were similar for extreme poverty rate but differed markedly for other indicators. For the 80/20 income ratio, the original Gini exhibited a stronger association (β = 0.027) than the normalized Gini (β = 0.002). In contrast, per capita income and the education indicator showed negative coefficients with the normalized Gini (β = − 0.016 and β = − 0.025, respectively), while being positive with the original index (β = 0.011 and β = 0.021).

**Table 3:** Bootstrap correlations between Gini indices (i.e., the original and normalized model) and socioeconomic indicators in Spain (2022)

Indicator	Metric	Index	Estimate	SE	CI 95%
Extreme poverty rate	*r*	Original Gini index	0.2886	0.0117	[0.2656, 0.3116]
		Normalized Gini index	0.6638	0.0075	[0.6491, 0.6785]
	*β*	Original Gini index	0.0349	0.0015	[0.0319, 0.0379]
		Normalized Gini index	0.0381	0.0006	[0.0371, 0.0392]
80/20 income ratio	*r*	Original Gini index	0.6761	0.0092	[0.6581, 0.6941]
		Normalized Gini index	0.1300	0.0116	[0.1073, 0.1527]
	*β*	Original Gini index	0.0273	0.0005	[0.0264, 0.0282]
		Normalized Gini index	0.0023	0.0002	[0.0018, 0.0027]
Per capita income	*r*	Original Gini index	0.1987	0.0135	[0.1723, 0.2250]
		Normalized Gini index	–0.5690	0.0085	[–0.5855, − 0.5524]
	*β*	Original Gini index	0.0115	0.0008	[0.0099, 0.0130]
		Normalized Gini index	–0.0162	0.0003	[–0.0167, − 0.0157]
Higher-to-lower education ratio	*r*	Original Gini index	0.1557	0.0127	[0.1307, 0.1806]
		Normalized Gini index	–0.3896	0.0099	[–0.4091, − 0.3702]
	*β*	Original Gini index	0.0213	0.0017	[0.0180, 0.0247]
		Normalized Gini index	–0.0252	0.0006	[–0.0264, − 0.0239]

SE, standard error; CI, confidence interval; r, Pearson correlation coefficient; β unstandardized coefficient from a linear regression of each indicator on the inequality index. Prior to regression, influential outliers were removed using Cook’s distance threshold. Log transformation was applied to the dependent variables to improve model fit and reduce heteroskedasticity, as assessed by AIC/BIC comparison. Standard errors and 95% confidence intervals were computed using the bootstrap distribution, resampling (*R* = 2000). All coefficients were statistically significant at the p-value < 0.001

## Discussion

In this study, we propose a simple modification of the original Gini index that incorporates the poverty rate as an offset. Combined with Bayesian spatial modelling, this approach improves its accuracy in areas of high deprivation and the precision of small-area estimates, easing territorial comparability. By applying this normalized Gini index across more than 8,000 Spanish municipalities, spatial patterns of inequality relative to local poverty levels—particularly in low-density or rural regions— are identified, that were overlooked by the original measure.

Our findings also contribute to the literature on spatial inequality and spatial dependence [22–25], which has highlighted the sensitivity of inequality measures to territorial aggregation and spatial autocorrelation. In contrast to these previous approaches, our framework allows for the estimation of inequality at a fine municipal scale (LAU-2) by explicitly incorporating the local poverty structure. This enables us to provide a more detailed and nuanced representation of territorial disparities, reducing the risk of local differences being smoothed out when analysed at more aggregated territorial scales.

Different strategies for incorporating the poverty rate into the model were explored in this study. The results indicate that the covariate specification achieved superior performance according to standard Bayesian model fit criteria (DIC, WAIC, and LPML). However, this improvement came at the cost of a substantial reduction in the variability of the predicted values, a feature that is central to the interpretation of territorial inequality. In contrast, the offset specification, although showing less optimal goodness-of-fit, retained a broader distribution of predicted values, more closely aligned with the observed variability, and therefore provided a more interpretable representation of spatial heterogeneity. For this reason, and given the objective of the study, the offset model was selected as the primary specification, while acknowledging that alternative formulations may offer improved statistical fit according to standard model fit criteria.

The results indicate that the explanatory capacity of the normalized Gini index is superior to that of the classical version. The normalized index exhibits stronger correlations and more stable regression coefficients across several relevant socioeconomic indicators, highlighting its enhanced sensitivity to structural dimensions of inequality. In contrast, the classical Gini index shows a stronger association only with the 80/20 income ratio, which is particularly sensitive to the extremes of the distribution. Rather than directly reproducing poverty gradients, these findings suggest that the normalized index captures territorial differences in inequality after accounting for the local poverty context. Furthermore, the standard errors associated with the normalized Gini are systematically lower, suggesting greater precision and robustness in its estimates.

Like other single summary statistics, the Gini index does not capture the distribution of income within each territory and does not accurately reflect the impact of redistributive policies [4–6]. This limitation affects its usefulness for the policy-making [6], especially when comparing territories with different demographic or economic structures. Prior research has emphasized the need to refine inequality measures using approaches that are more sensitive to the internal distribution of income [38] or by conditioning the Gini coefficient [39]. Our model addresses these limitations by incorporating the poverty rate—a widely available and internationally recognized indicator— as an offset, thereby allowing inequality to be interpreted relative to the local poverty context and improving comparability across territories with heterogeneous sizes and socioeconomic characteristics.

The Gini index is also highly sensitive to outliers, which particularly affect calculations in small areas or low populated areas, where results may be unstable and unrepresentative [6–8]. Our model addresses these limitations through Bayesian spatial modelling, which smooths out outliers and produces more stable and consistent estimates [12–14]. In Italy, over the past few years, researchers have studied inequality by applying the Gini index to EU-SILC data [40, 41]. Using Bayesian models, these studies have managed to reduce the variability of the results, especially in areas with few responses. In France, Gautier [42] also found that modelling inequality with Bayesian approaches allowed for a more accurate capture of the income distribution. However, none of them have calculated the Gini index for areas with fewer than 100 inhabitants in a way that allows for consistent readings. This methodological advantage may be useful for policymakers seeking to implement targeted interventions in small municipalities, where instability in estimates may hinder evidence-based planning.

While this study focused on Spain, the proposed method is broadly applicable to other settings, since the poverty rate is regularly reported by national statistical institutes or international organizations [26]. In addition, the method can be adapted to different territorial levels, since the poverty threshold can be calculated specifically for each area. This would allow, for instance, the comparison of inequalities between countries using harmonized thresholds, as well as the application of the method to more disaggregated administrative units. Additionally, the model can be complemented with other contextual variables—such as employment rates, education, or housing costs—to further refine estimates and improve the interpretation of territorial inequality.

Applying this method to Spain revealed notable shifts in inequality patterns. According to the original Gini index, the Balearic Islands and the Community of Madrid had the highest proportion of municipalities with very high inequality (Q4), while Extremadura and Galicia recorded the lowest levels. After normalization, Madrid, the Balearic Islands, Catalonia, and the Basque Country showed a reduced proportion of municipalities with high inequality, whereas Extremadura emerged among the three regions with inequality levels higher than expected given their poverty context, along with Andalusia and Murcia. This shift in the patterns is partially consistent with previous studies [43–48], which tend to report lower inequality levels in Madrid and the northeast of Spain and the highest levels in the South. Other studies have focused on cities and urban areas [46–48], highlighting significant interurban inequalities in Catalonia and Madrid. Our results before normalization reflect this trend: urban and highly dense areas exhibit greater inequality than rural areas. However, the normalized results reversed this pattern, suggesting that, after accounting for poverty levels, rural or low-density areas show higher relative inequality.

Several limitations must be acknowledged when interpretating our findings. First, although our method addresses some of the Gini index limitations, it remains a unidimensional measure and should be interpreted alongside other indicators to provide a more nuanced understanding of income distribution [14]. Second, the approach was only applied in the Spanish context. Although the method is generalizable, further validation in other settings with different income structures and poverty thresholds is needed. Third, it should be noted that, when making comparisons, no adjustment was made for consumption levels across different areas, which can lead to partial interpretations, especially regarding saving capacity. Moreover, the absence of the informal economy or unpaid work - a limitation inherent to the official data source - could further affect our findings. Finally, despite providing aggregated values at a small-area level, Spanish population dense municipalities, such as Madrid or Barcelona, may present marked internal inequalities across districts or neighbourhoods, which could be captured in lower scale analyses. Further studies disaggregating the territorial dimension in greater detail and incorporating additional variables such as cost of living and saving capacity, are warranted to improve the precision of comparisons between territories and to refine the interpretation of inequality relative to local poverty levels.

## Conclusions

This study proposes a normalized Gini index that incorporates the poverty rate and Bayesian spatial modelling to improve the measurement of income inequality at small-area levels. Applied to Spain, this approach has revealed inequality patterns overlooked by the original index, particularly in highly deprived or rural regions. Its simplicity and reliance on widely available data make it easily applicable in other settings, enhancing comparability across territories and providing a more accurate basis for local redistributive policies.

## Electronic Supplementary Material

Below is the link to the electronic supplementary material.

Supplementary Material 1

## Data Availability

Socio-economic municipal-level data for 2022 was obtained from the Household Income Distribution Atlas (HIDA), which is openly provided by Spanish National Statistics Institute. Available here: https://www.ine.es/dyngs/Prensa/es/ADRH2022.htm.

## Abbreviations

CI: Credibility interval
DIC: Deviance information criterion
HIDA: Household income distribution atlas
LPML: Logarithm of the pseudo marginal likelihood
SDG: Sustainable development goals
WAIC: Watanabe–Akaike information criterion
