# Supplementary material for: Beyond the Gini index: a poverty-normalized spatial Bayesian model for assessing territorial inequality
Source: Int J Equity Health. 2026 May 26;25:174. doi: 10.1186/s12939-026-02890-3 (PMC13397631; doi:10.1186/s12939-026-02890-3)
Supplement: Supplementary file 1 — Supplementary Material 1 [file 12939_2026_2890_MOESM1_ESM.docx]

**Beyond the Gini index: a poverty-normalized spatial Bayesian model for assessing territorial inequality.**

Xavier Perafita, Marta Solans, Laura Vilà-Quintana,

Maria Antònia Barceló, and Marc Saez.

**Supplementary material**

# Content

[Figure S1. The Lorenz Curve. 3](#_Toc227745591)

[Figure S2. Comparison of observed and imputed distributions across different imputation methods. 5](#_Toc227745592)

[Figure S3. Quantile–quantile plots comparing observed and imputed poverty rate values under the final PMM model. 6](#_Toc227745593)

[Figure S4. Observed and imputed distributions of the poverty rate using predictive mean matching (PMM). 6](#_Toc227745594)

[Figure S5. Comparison of the data distribution the original Gini index and its normalized version across the 16 autonomous communities (NUTS-2) in Spain (2022). 7](#_Toc227745595)

[Figure S6. Geographical representation of the normalized Gini index and the difference in relation to the original index across the 6 NUTS-1 of Spain in 2022. 13](#_Toc227745596)

[Figure S7A. Distribution of the percentage of extreme poverty across municipalities in Spain (2022). 14](#_Toc227745597)

[Figure S7B. Distribution of the percentage of gross income per capita across municipalities in Spain (2022). 15](#_Toc227745598)

[Figure S7C. Distribution of the percentage of higher-to-basic education attainment ratio across municipalities in Spain (2022) 16](#_Toc227745599)

[Table S1. Model comparison of the three alternative Gini indices based on Bayesian information criteria and posterior predictive summaries. 17](#_Toc227745600)

[Table S2. Width of the 95% credible intervals for the predicted Gini index across the three models. 17](#_Toc227745601)

[Table S3. Bootstrap correlations between Gini indices (original and alternative models incorporating poverty through offsets, covariates, or weights) and socioeconomic indicators in Spain (2022). 18](#_Toc227745602)

[Table S4. Missing data patterns for Gini index and poverty rate across the 8,138 Spanish municipalities (including Canary Islands). 19](#_Toc227745603)

[Table S5. Comparison of observed and imputed poverty rate statistics 19](#_Toc227745604)

[Table S6. Sensitivity analysis comparing the imputed-data model and the complete-case model. 19](#_Toc227745605)

[Table S7. Summary statistics of the original Gini and the normalized Gini index at NUTS-3 level in Spain (2022). 20](#_Toc227745607)


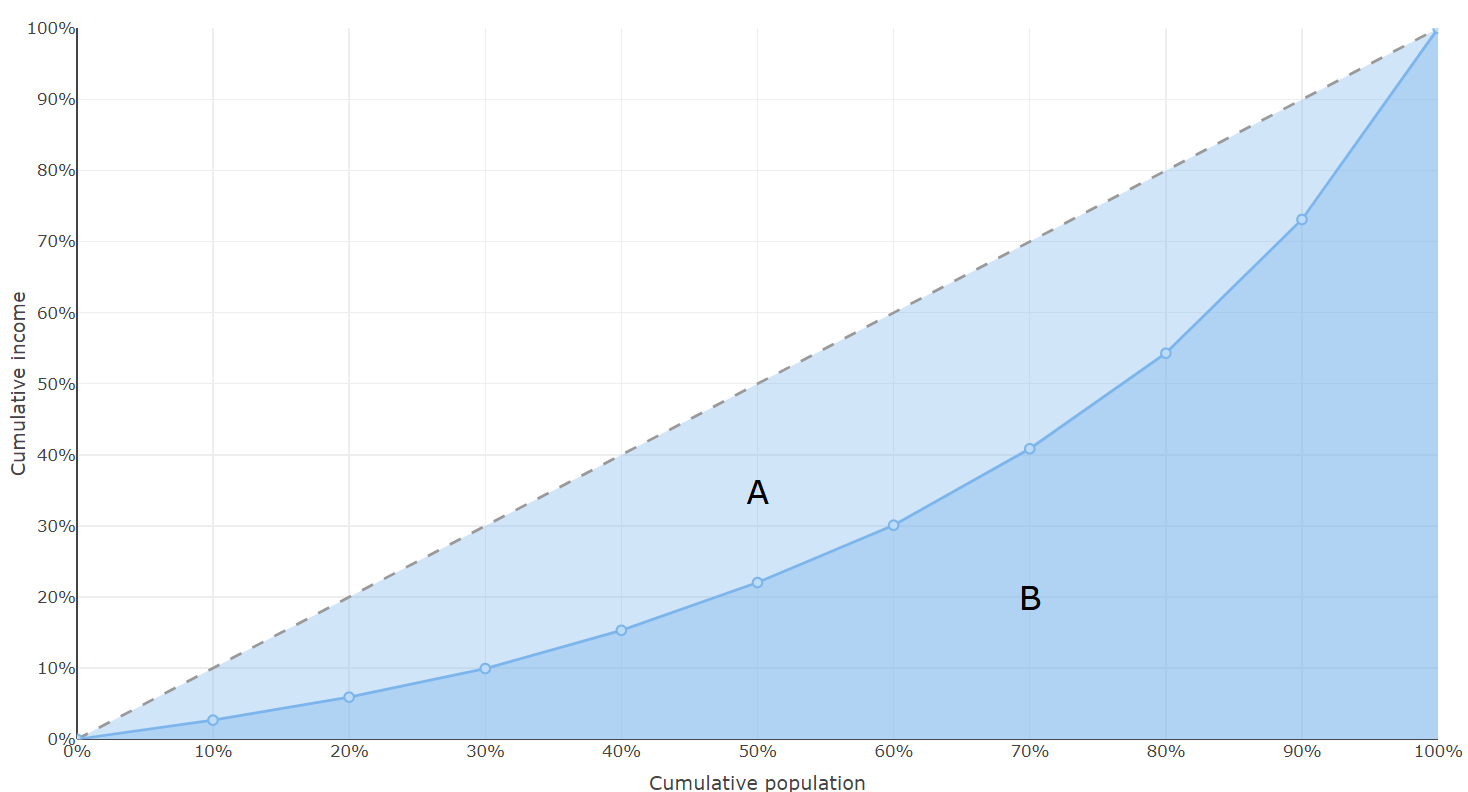


# **Figure S1**. The Lorenz Curve. The Gini index is calculated as the ratio of the area between the perfect equality line and the Lorenz curve (*A*) divided by the total area under the perfect equality line (*A* + *B*).

**A. Deterministic linear regression imputation**


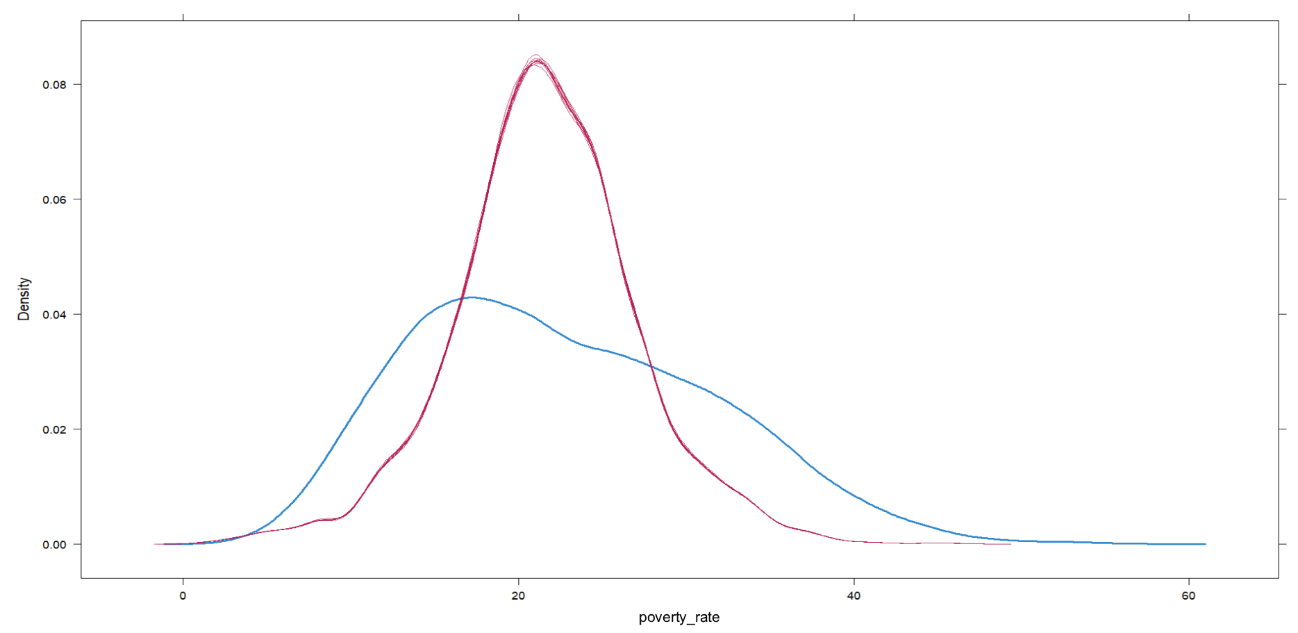


**B. Predictive mean matching (PMM)**


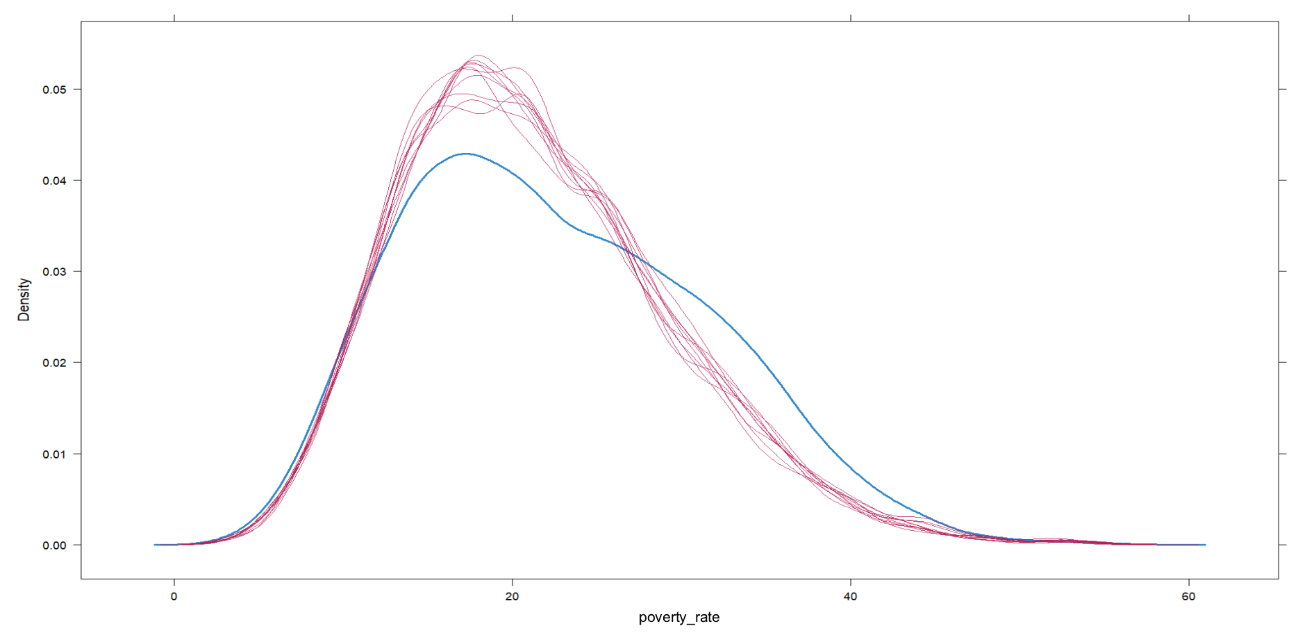


**C. Linear regression without residual error**


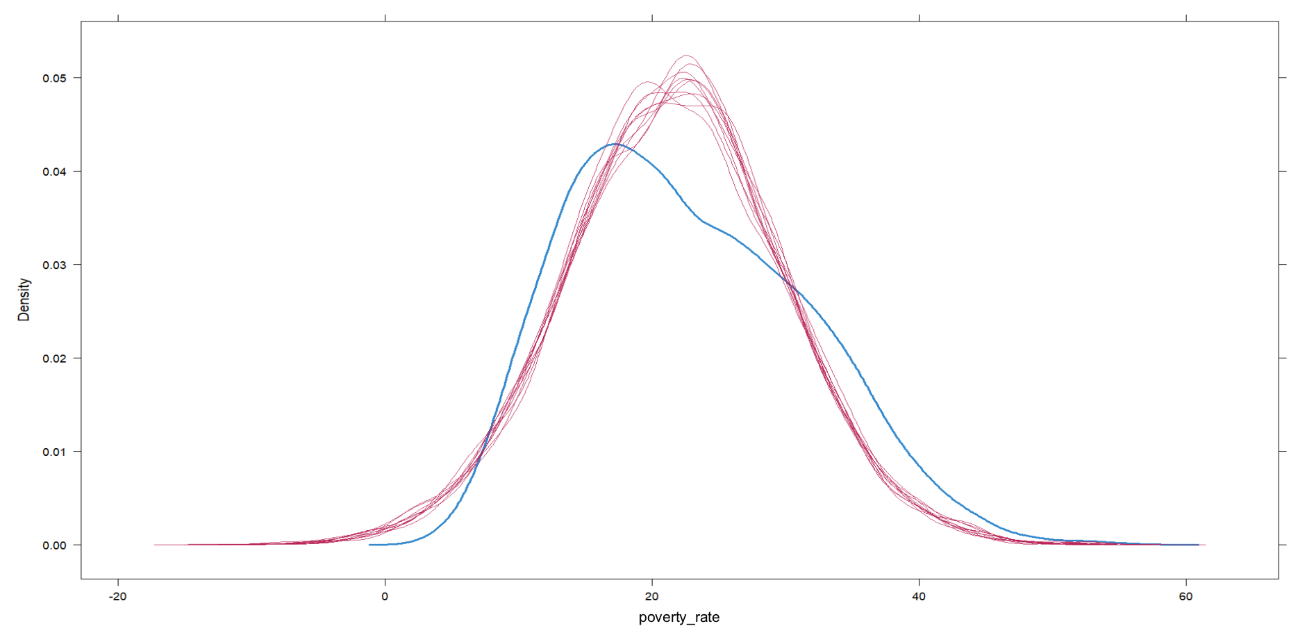


**D. Bayesian linear regression**


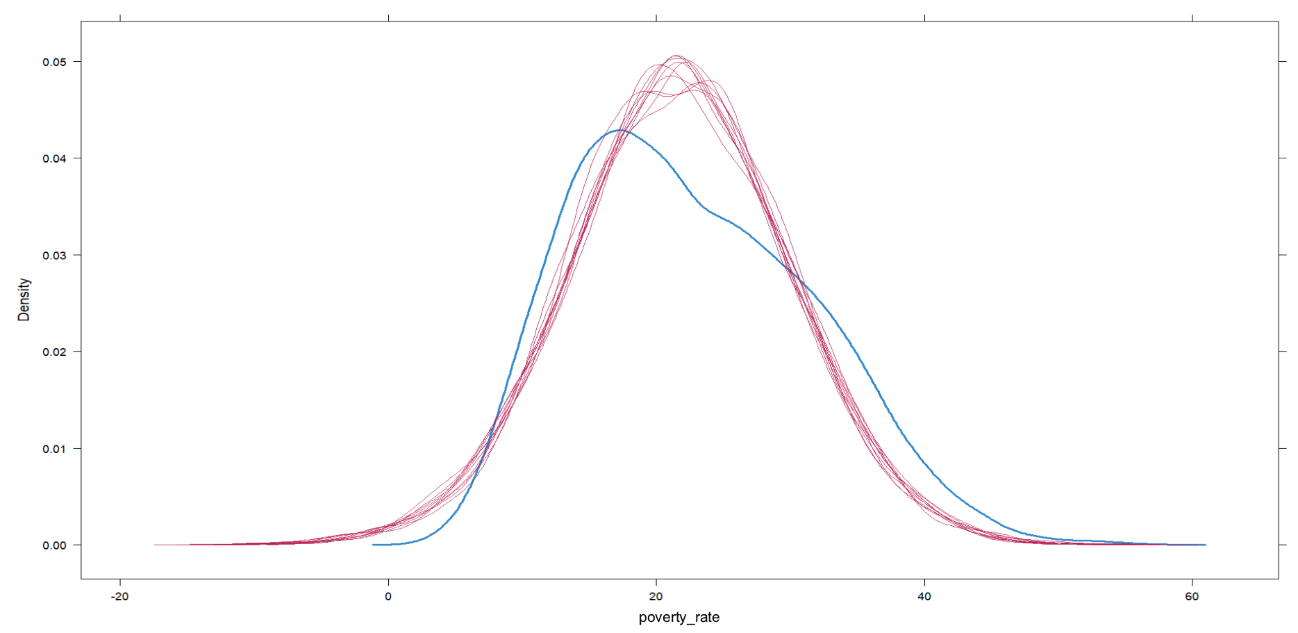


**E. Classification and regression trees (CART)**


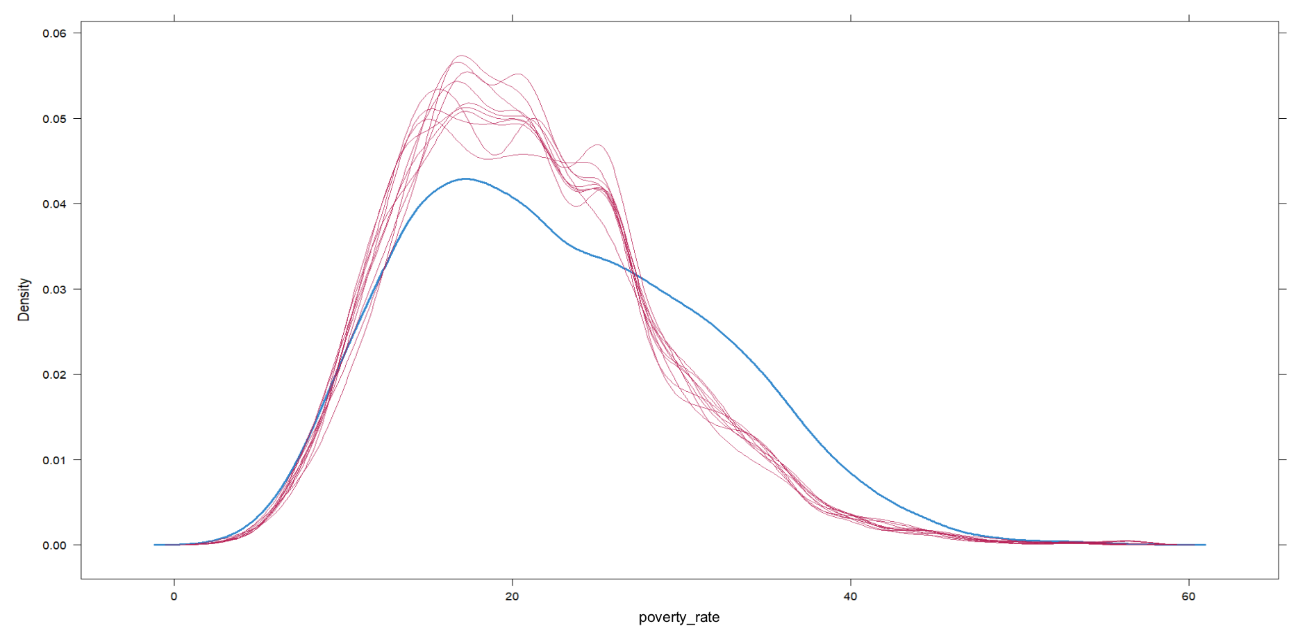


# **Figure S2**. Comparison of observed and imputed distributions across different imputation methods. Density plots comparing observed poverty rate values (blue line) and imputed datasets (red lines) across different imputation methods. All methods were implemented using multiple imputation by chained equations with 10 imputations and 5 iterations.


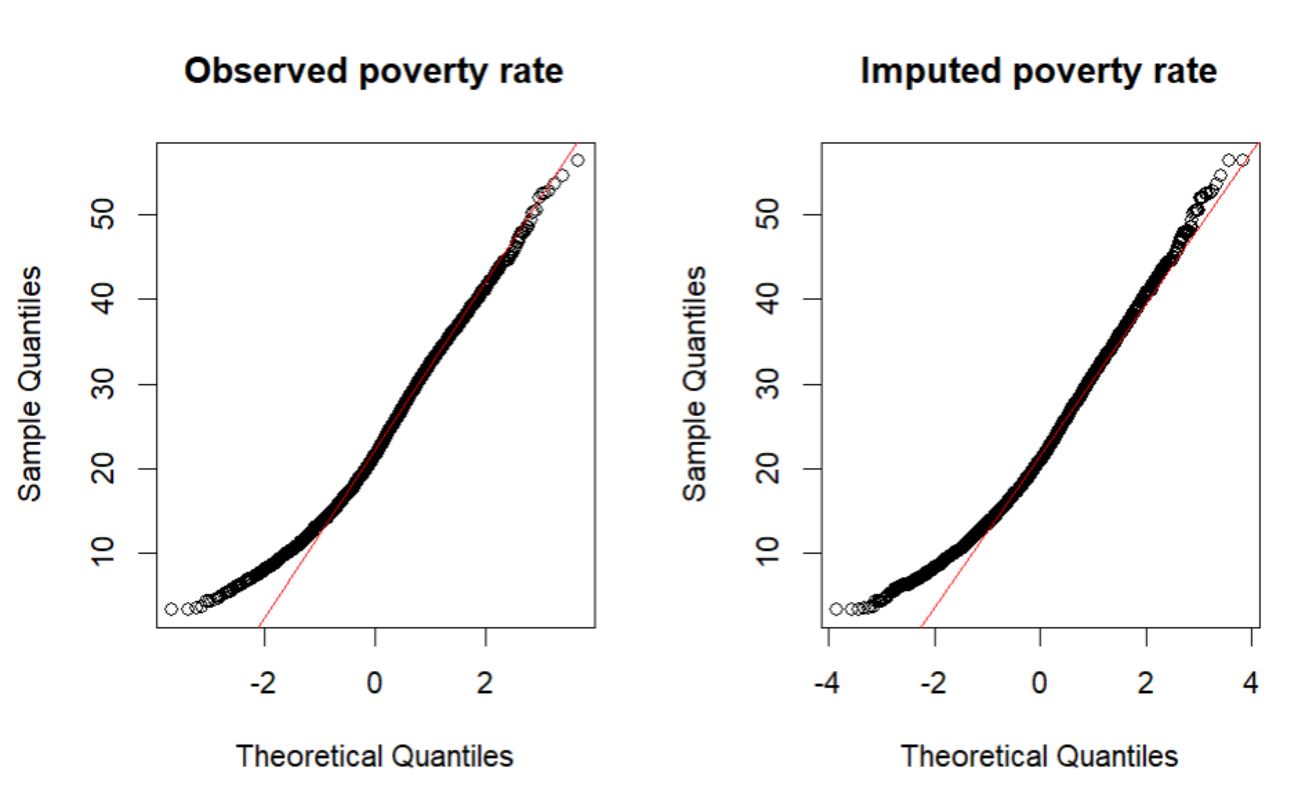


# **Figure S3**. Quantile–quantile plots comparing observed and imputed poverty rate values under the final PMM model.


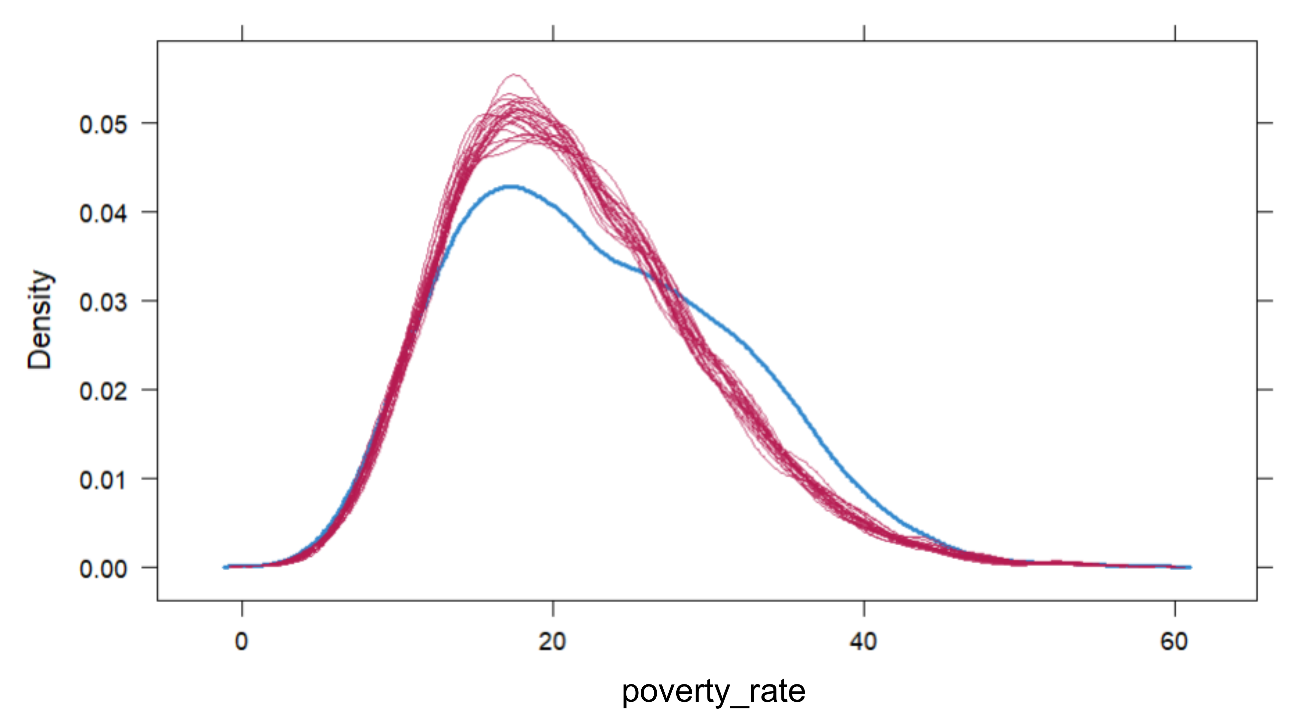


# **Figure S4**. Observed and imputed distributions of the poverty rate using predictive mean matching (PMM). Density plots comparing observed poverty rate values (blue line) and imputed datasets (red lines) obtained using the final predictive mean matching (PMM) model. The model was implemented with 25 imputations and 10 iterations.


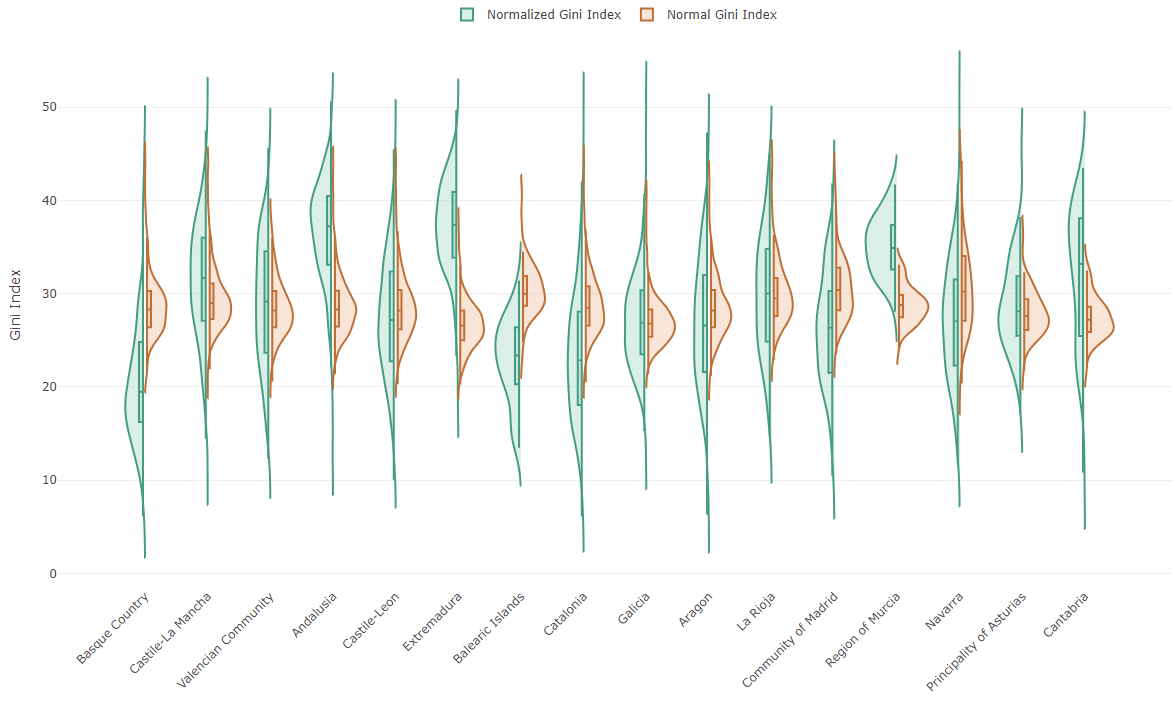


# **Figure S5**. Comparison of the data distribution the original Gini index and its normalized version across the 16 autonomous communities (NUTS-2) in Spain (2022). Ceuta and Melilla were excluded from the representation.

**NUTS1 – ES1 (Northwest)**


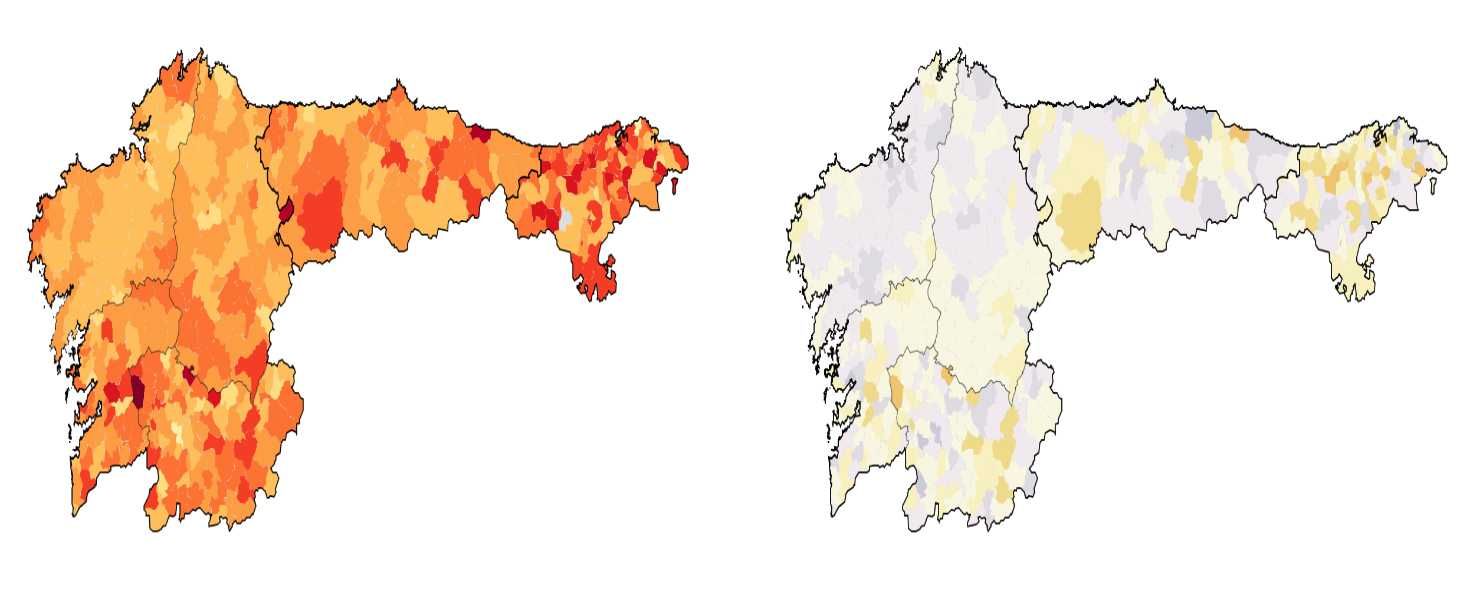


**NUTS1 – ES2 (Northeast)**


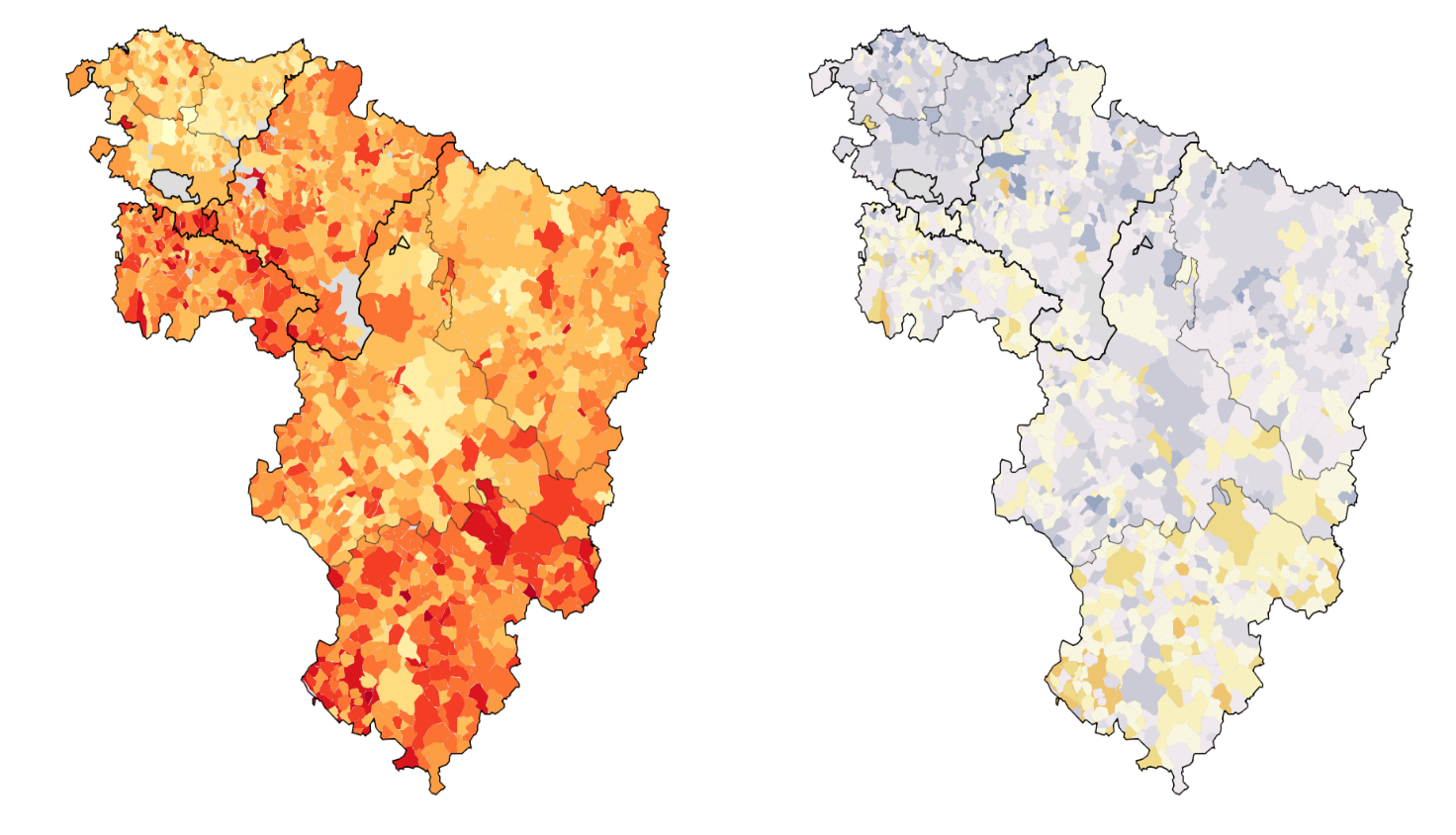


**NUTS1 – ES3 (Community of Madrid)**


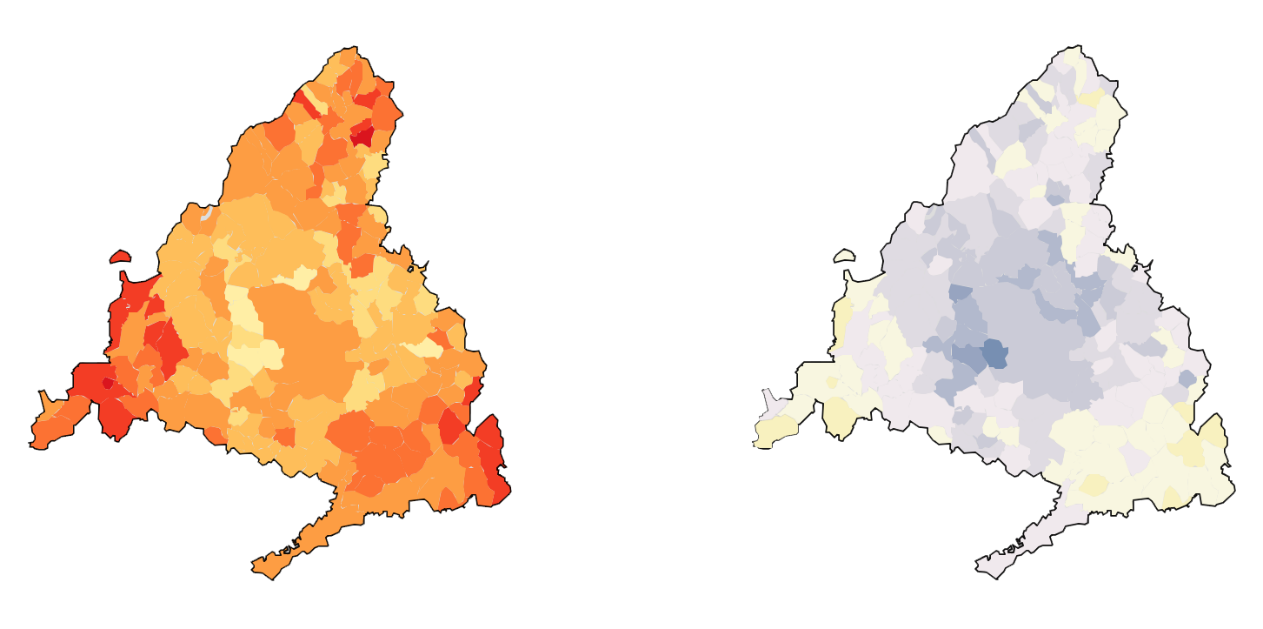


**NUTS1 – ES4 (Centre)**


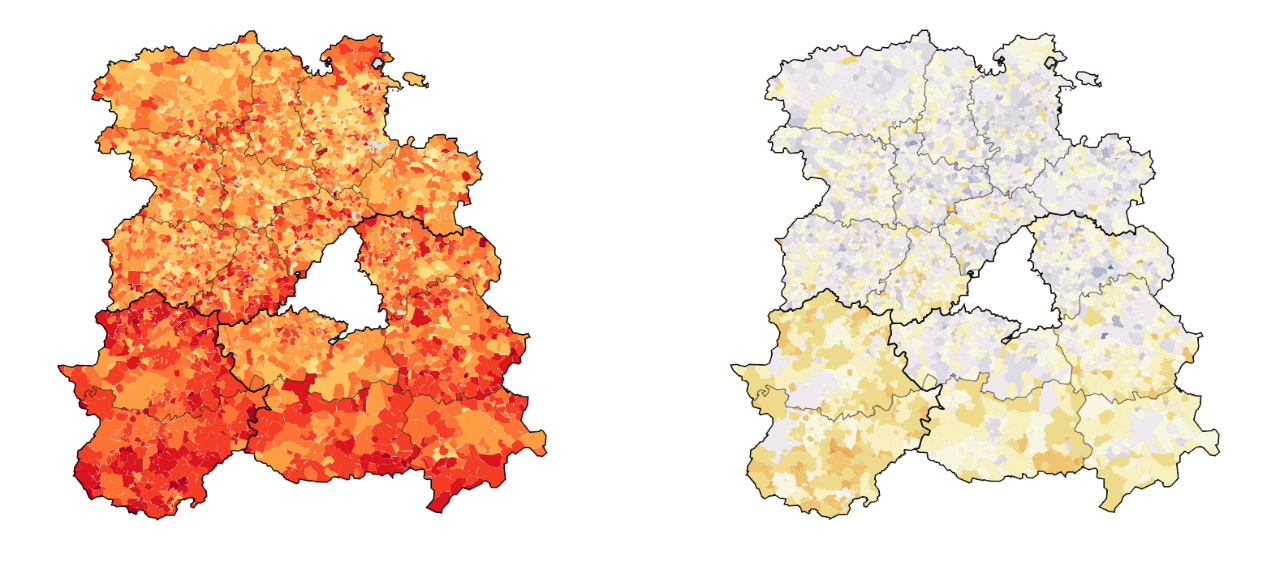


**NUTS1 – ES5 (East)**


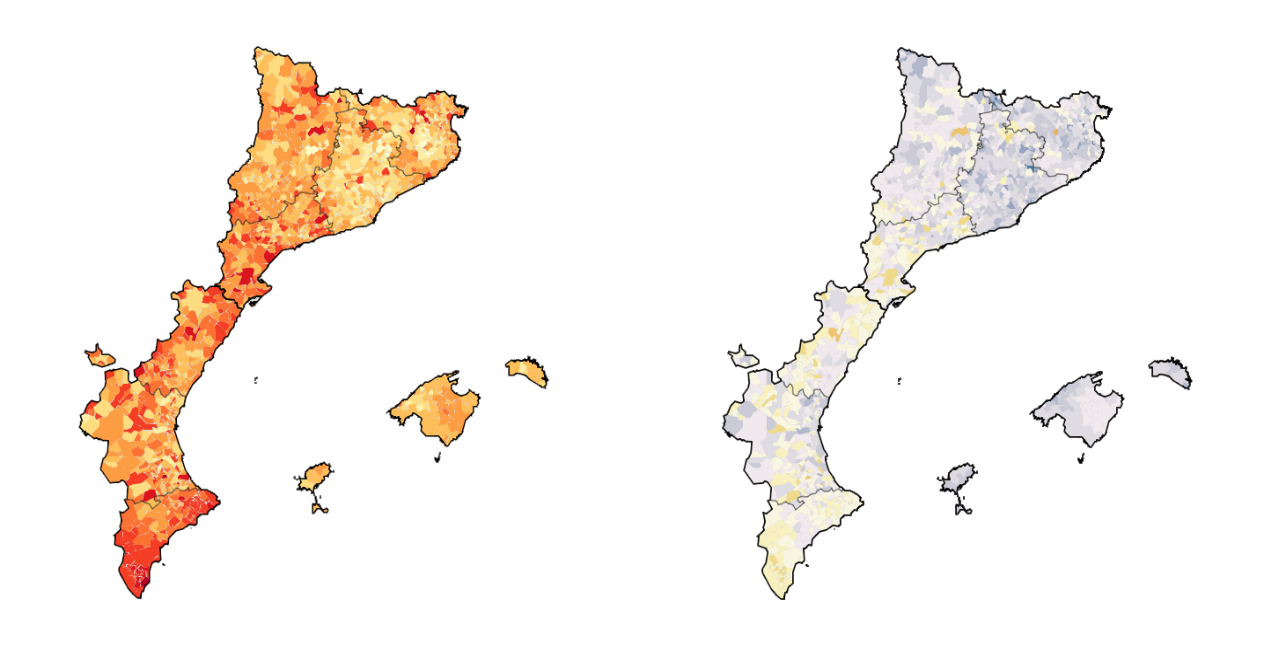


**NUTS1 – ES6 (South)**


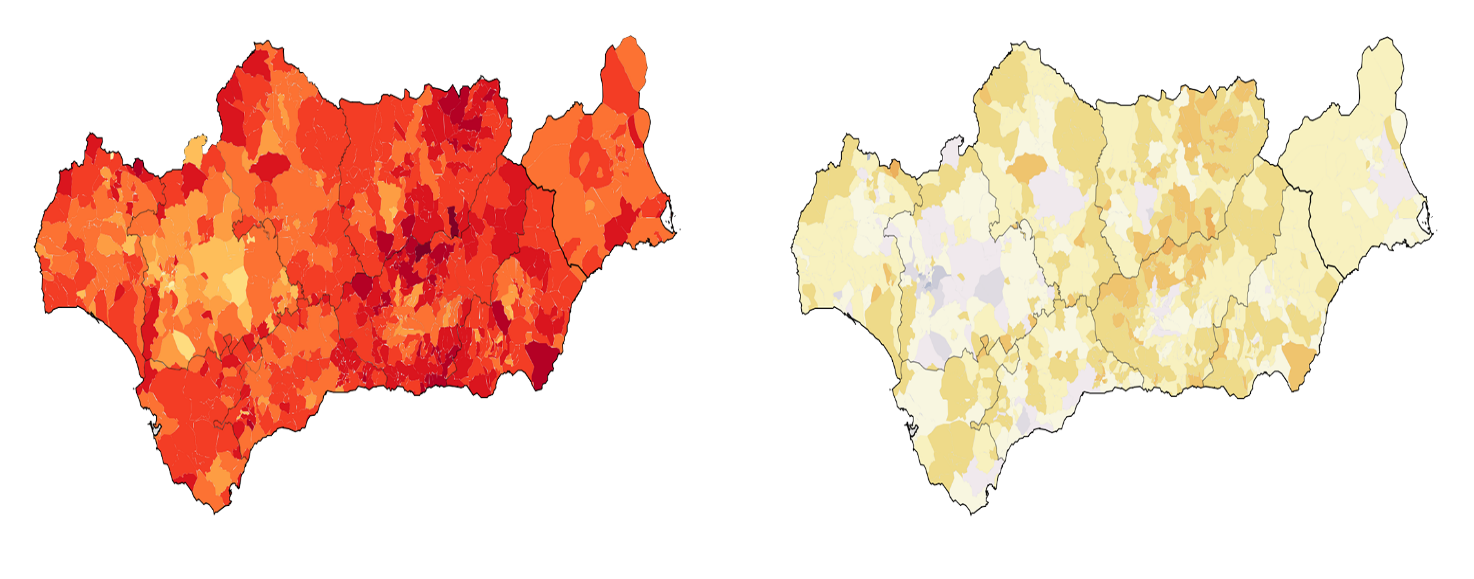


# **Figure S6**. Geographical representation of the normalized Gini index and the difference in relation to the original index across the 6 NUTS-1 of Spain in 2022.


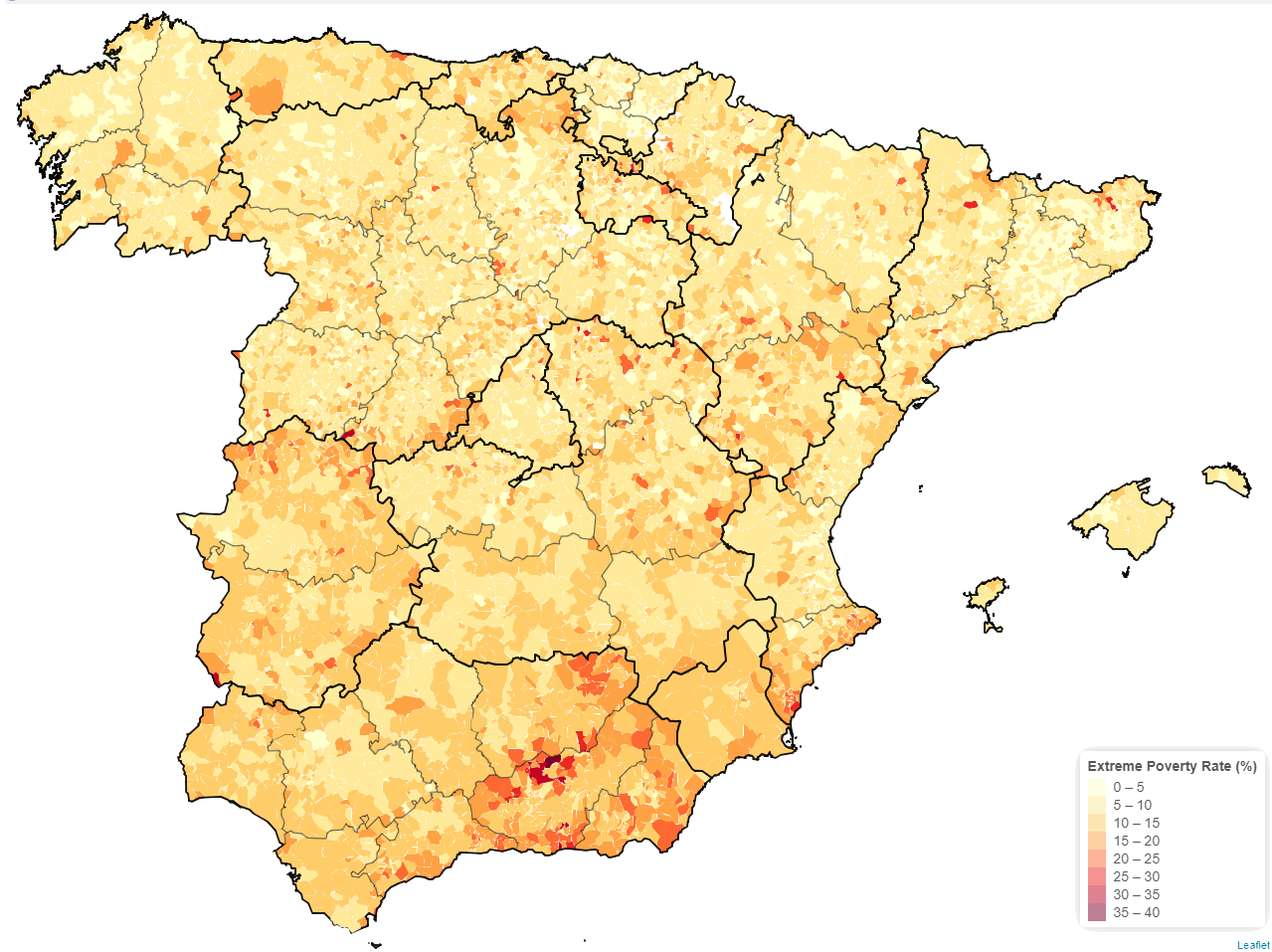


# **Figure S7A**. Distribution of the percentage of extreme poverty across municipalities in Spain (2022).


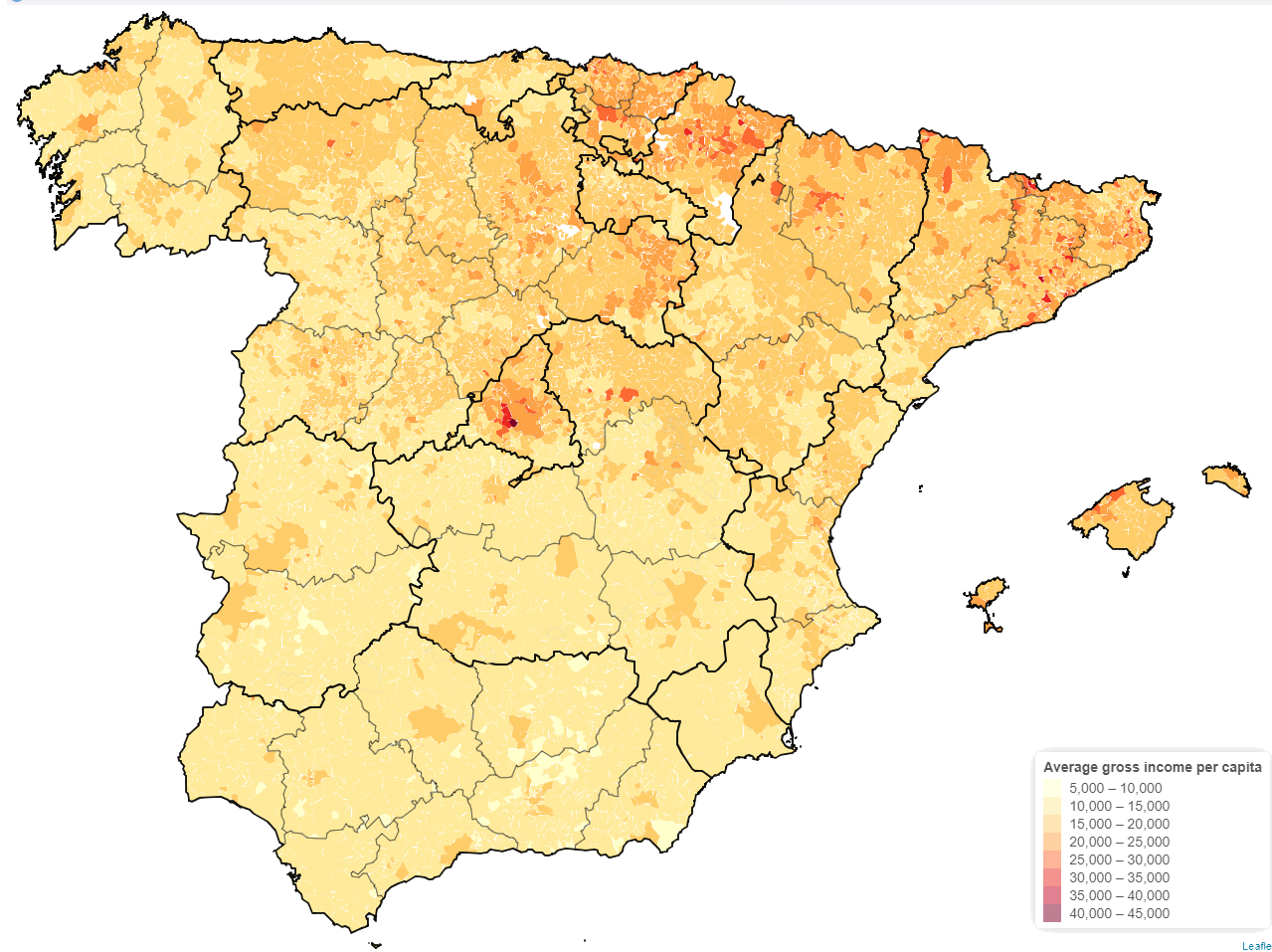


# **Figure S7B**. Distribution of the percentage of gross income per capita across municipalities in Spain (2022).


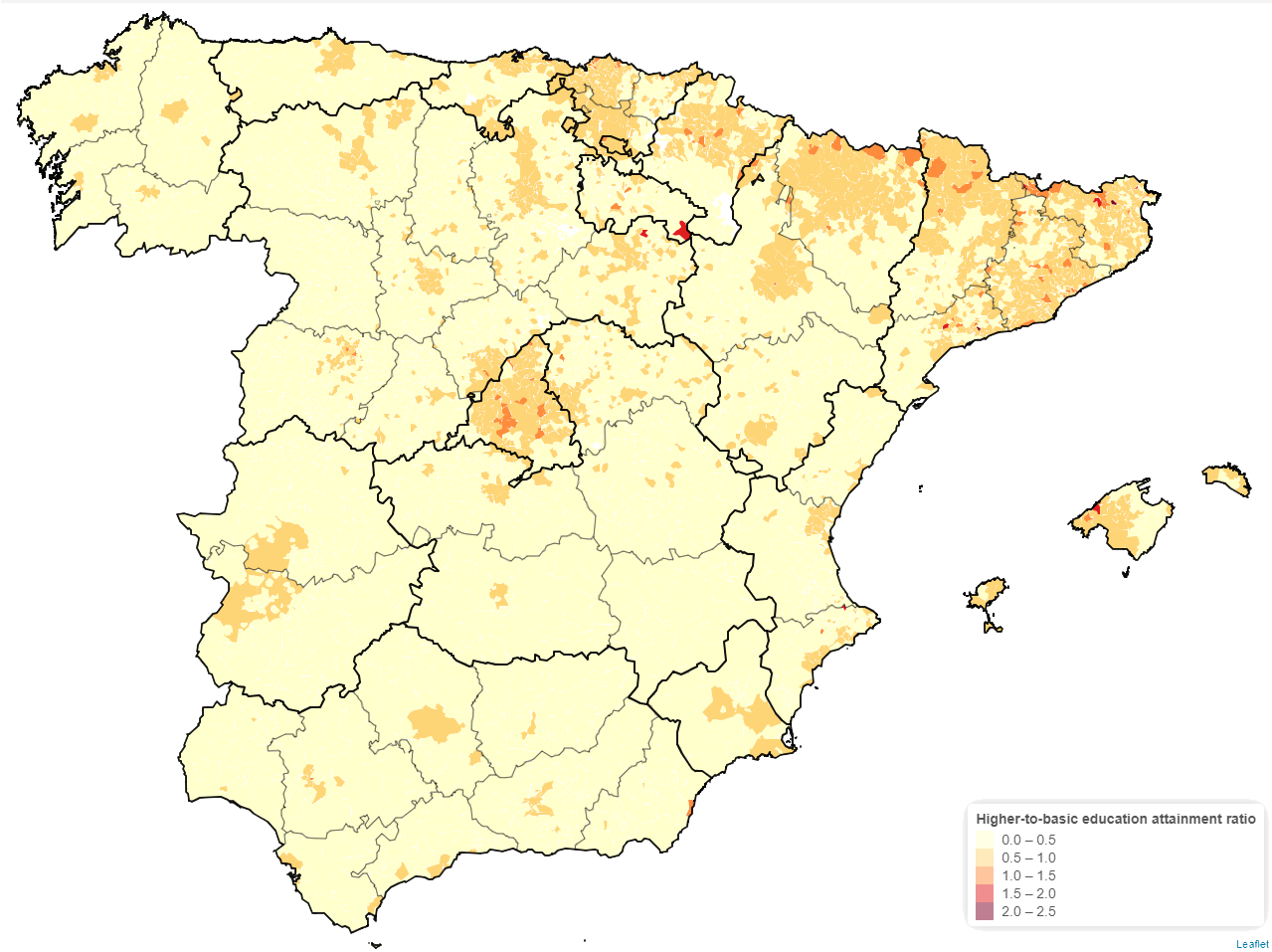


# **Figure S7C**. Distribution of the percentage of higher-to-basic education attainment ratio across municipalities in Spain (2022).

# **Table S1**. Model comparison of the three alternative Gini indices based on Bayesian information criteria and posterior predictive summaries.

| **Model comparisons** | **Offset^1^** | **Covariate^2^** | **Weights^3^** |
| --- | --- | --- | --- |
| DIC | -17,086.94 | -31,944.18 | -6,938.44 |
| Mean deviance | -17,195.97 | -32,019.35 | -6,942.48 |
| pD | 109.03 | 75.18 | 4.04 |
| WAIC | -17,089.81 | -31,940.37 | -6,943.36 |
| LPML | 8,544.36 | 15,969.42 | 3,470.61 |
| Predicted value range | 0.06-0.52 | 0.27-0.31 | 0.29-0.29 |
| SD of predicted values | 0.0777 | 0.0057 | 0.0000 |
| Mean predicted value | 0.2869 | 0.2871 | 0.2894 |
| Mean observed Gini index | 0.2871 | 0.2871 | 0.2871 |
| Difference | -0.0002 | 0.0001 | 0.0023 |

DIC, deviance information criterion; LPML, log pseudomarginal likelihood; pD, effective number of parameters ; SD, standard deviation WAIC, Watanabe Akaike information criterion.

^1^Includes a log-offset term for the poverty rate.

^2^Explicitly includes the poverty rate as a fixed effect.

^3^Uses observational weights proportional to the poverty rate.

# **Table S2**. Width of the 95% credible intervals for the predicted Gini index across the three models.

| **Model** | **95% credible interval width** | | |
| --- | --- | --- | --- |
|  | *Mean* | *Min* | *Max* |
| Offset^1^ | 0.0387 | 0.0114 | 0.0876 |
| Covariate^2^ | 0.0137 | 0.0131 | 0.0598 |
| Weights^3^ | 0.0058 | 0.0057 | 0.0250 |

^1^Includes a log-offset term for the poverty rate.

^2^Explicitly includes the poverty rate as a fixed effect.

^3^Uses observational weights proportional to the poverty rate.

# **Table S3**. Bootstrap correlations between Gini indices (original and alternative models incorporating poverty through offsets, covariates, or weights) and socioeconomic indicators in Spain (2022).

| **Model^1^** | **Correlation** | **Bias** | **Bootstrap** | | **CI** | |
| --- | --- | --- | --- | --- | --- | --- |
|  |  |  | *SE* | *Mean* | *2,5* | *97,5* |
| **Extreme poverty rate** | | | | | | |
| Original | 0.2886 | -0.0000 | 0.0121 | 0.2886 | 0.2648 | 0.3125 |
| Offset | 0.6638 | 0.0003 | 0.0076 | 0.6641 | 0.6489 | 0.6786 |
| Covariate | 0.6925 | 0.0002 | 0.0080 | 0.6927 | 0.6769 | 0.7081 |
| Weighted | 0.2764 | 0.0046 | 0.0259 | 0.2810 | 0.2256 | 0.3272 |
| **80/20 income ratio** | | | | | | |
| Original | 0.6761 | 0.0004 | 0.0090 | 0.6765 | 0.6585 | 0.6938 |
| Offset | 0.1300 | 0.0005 | 0.0114 | 0.1305 | 0.1076 | 0.1523 |
| Covariate | 0.1596 | 0.0005 | 0.0120 | 0.1601 | 0.1361 | 0.1830 |
| Weighted | 0.5682 | 0.0116 | 0.0655 | 0.5798 | 0.4399 | 0.6966 |
| **Average gross income per capita** | | | | | | |
| Original | 0.1987 | -0.0000 | 0.0133 | 0.1986 | 0.1727 | 0.2247 |
| Offset | -0.5690 | -0.0001 | 0.0085 | -0.5691 | -0.5857 | -0.5523 |
| Covariate | -0.5435 | -0.0001 | 0.0087 | -0.5436 | -0.5605 | -0.5265 |
| Weighted | 0.1375 | 0.0044 | 0.0245 | 0.1418 | 0.0894 | 0.1855 |
| **Higher-to-lower education ratio** | | | | | | |
| Original | 0.1557 | 0.0001 | 0.0127 | 0.1558 | 0.1309 | 0.1805 |
| Offset | -0.3896 | 0.0001 | 0.0099 | -0.3895 | -0.4091 | -0.3701 |
| Covariate | -0.3642 | 0.0001 | 0.0094 | -0.3642 | -0.3828 | -0.3457 |
| Weighted | 0.1155 | 0.0031 | 0.0200 | 0.1186 | 0.0763 | 0.1548 |

CI, confidence interval; SE, standard error.

^1^The four models compared include: the original Gini index (Original) and the three alternative approaches that include the poverty rate as a log-offset term (Offset), as a fixed effect (Covariate), or uses observational weights proportional to the poverty rate (Weighted).

# **Table S4**. Missing data patterns for Gini index and poverty rate across the 8,138 Spanish municipalities (including Canary Islands).

|  | **Poverty rate observed** | **Poverty rate missing** | **Total** |
| --- | --- | --- | --- |
| **Gini observed** | 3,804 | 2,968 | 6,772 |
| **Gini missing** | 329 | 1,037 | 1,366 |
| **Total** | 4,133 | 4,005 | 8,138 |

# **Table S5.** Comparison of observed and imputed poverty rate statistics.

| **Statistic** | **Observed** | **Imputed** |
| --- | --- | --- |
| Mean | 22.66 | 22.04 |
| Median | 21.60 | 21.00 |
| Q1 | 15.70 | 15.60 |
| Q3 | 29.10 | 27.70 |
| Min | 3.40 | 3.40 |
| Max | 56.40 | 56.40 |
| Skewness | 0.41 | 0.50 |
| Kurtosis | 2.61 | 2.87 |

# **Table S6.** Sensitivity analysis comparing the imputed-data model and the complete-case model.

| **Analysis** | **N municipalities** | **Mean fitted normalized Gini** | **SD** | **Minimum** | **Maximum** |
| --- | --- | --- | --- | --- | --- |
| Main analysis (imputed data) | 8,043 | 28.69 | 7.77 | 6.13 | 51.50 |
| Complete-case analysis | 6,684 | 28.56 | 7.84 | 6.04 | 51.16 |

N, total number; SD, standard deviation.

# **Table S7.** Summary statistics of the original Gini and the normalized Gini index at NUTS-3 level in Spain (2022).

| **NUTS-3** | **N** | **Original Gini index** | | | **Normalized Gini index** | | |
| --- | --- | --- | --- | --- | --- | --- | --- |
|  |  | *Mean (SD)* | *Median (Q1-Q3)* | *Min-Max* | *Mean (SD)* | *Median (Q1-Q3)* | *Min-Max* |
| A Coruña | 93 | 26.13 (2.16) | 26.13 (24.40-27.10) | 22.4-34.2 | 24.83 (3.71) | 24.83 (22.38-27.17) | 15.28-34.04 |
| Lugo | 67 | 26.66 (2.63) | 26.66 (25.20-28.15) | 22.7-40.7 | 26.77 (4.56) | 26.77 (24.08-28.91) | 16.23-46.02 |
| Ourense | 92 | 27.96 (1.81) | 27.96 (26.80-28.90) | 24.9-34.4 | 28.89 (6.55) | 28.89 (24.72-32.11) | 12.46-51.49 |
| Pontevedra | 61 | 26.84 (2.42) | 26.84 (25.20-28.30) | 21.4-34.4 | 28.67 (5.09) | 28.67 (25.43-32.03) | 17.91-40.42 |
| Asturias | 78 | 27.78 (2.36) | 27.78 (26.10-29.40) | 21.8-36.3 | 28.56 (4.96) | 28.56 (25.49-31.87) | 17.18-45.70 |
| Cantabria | 102 | 27.42 (2.30) | 27.42 (25.92-28.60) | 21.7-33.6 | 31.91 (7.2) | 31.91 (25.57-37.95) | 10.82-43.50 |
| Álava | 51 | 30.90 (3.50) | 30.90 (27.95-33.25) | 25.5-38.8 | 26.26 (9.72) | 26.26 (18.92-33.64) | 8.62-45.69 |
| Gipuzkoa | 88 | 27.33 (3.23) | 27.33 (25.58-28.90) | 21.4-44.2 | 17.90 (3.86) | 17.90 (15.81-20.26) | 6.13-27.96 |
| Bizkaia | 112 | 28.62 (2.52) | 28.62 (26.68-30.22) | 22.1-35.3 | 21.10 (6.85) | 21.10 (16.62-25.64) | 7.80-43.39 |
| Community of Navarre | 272 | 30.91 (4.87) | 30.91 (27.10-34.02) | 20.4-44.2 | 27.07 (6.57) | 27.07 (22.31-31.52) | 11.71-51.49 |
| La Rioja | 174 | 29.94 (3.32) | 29.94 (27.60-31.70) | 22.9-44.2 | 29.92 (6.70) | 29.92 (24.9-34.79) | 14.76-45.05 |
| Huesca | 202 | 28.15 (2.75) | 28.15 (26.33-29.80) | 21.2-36 | 24.30 (6.40) | 24.30 (20.37-28.38) | 6.31-44.79 |
| Teruel | 236 | 28.94 (3.66) | 28.94 (26.60-31.22) | 20.3-42.5 | 31.50 (6.42) | 31.50 (27.51-36.29) | 12.90-47.30 |
| Zaragoza | 293 | 28.46 (3.40) | 28.46 (26.20-30.40) | 21.3-42.5 | 24.74 (6.49) | 24.74 (19.94-29.29) | 8.78-40.48 |
| Madrid | 179 | 30.69 (3.41) | 30.69 (28.25-32.80) | 23.6-42.6 | 26.21 (6.18) | 26.21 (21.56-30.25) | 10.51-41.83 |
| Ávila | 248 | 28.99 (3.51) | 28.99 (26.80-30.75) | 20.7-44.2 | 31.88 (6.70) | 31.88 (26.60-36.65) | 13.34-44.31 |
| Burgos | 371 | 28.39 (3.55) | 28.39 (25.90-30.40) | 20.3-42.6 | 25.98 (7.01) | 25.98 (20.93-30.76) | 11.69-47.34 |
| León | 211 | 27.44 (2.94) | 27.44 (25.50-29.10) | 20.4-40.1 | 26.56 (5.47) | 26.56 (22.75-30.19) | 12.89-45.21 |
| Palencia | 191 | 28.22 (3.16) | 28.22 (26.15-30.05) | 21.1-37.7 | 26.91 (6.24) | 26.91 (22.42-31.51) | 10.96-40.61 |
| Salamanca | 362 | 28.51 (3.56) | 28.51 (26.20-30.37) | 20.3-44.2 | 28.08 (5.96) | 28.08 (23.82-32.24) | 10.80-45.46 |
| Segovia | 209 | 29.38 (3.94) | 29.38 (26.70-31.70) | 20.7-44.2 | 28.85 (7.11) | 28.85 (23.89-34.02) | 10.82-47.79 |
| Soria | 183 | 28.55 (3.56) | 28.55 (26.30-30.40) | 20.5-41.2 | 25.21 (6.10) | 25.21 (20.55-29.30) | 10.06-41.33 |
| Valladolid | 225 | 28.9 (3.66) | 28.90 (26.50-31.00) | 20.3-44.2 | 25.86 (6.89) | 25.86 (20.80-30.62) | 10.83-45.49 |
| Zamora | 248 | 28.45 (3.20) | 28.45 (26.40-30.10) | 20.3-44.2 | 27.79 (6.00) | 27.79 (23.64-32.40) | 13.33-41.99 |
| Albacete | 87 | 29.01 (2.24) | 29.01 (27.65-29.80) | 25.0-37.2 | 34.80 (4.97) | 34.80 (32.6-38.11) | 16.94-43.74 |
| Ciudad Real | 102 | 28.14 (20) | 28.14 (26.63-29.58) | 24.4-33.6 | 35.55 (5.57) | 35.55 (32.33-39.47) | 20.59-46.94 |
| Cuenca | 238 | 29.56 (3.41) | 29.56 (27.20-31.40) | 22.8-44.2 | 33.15 (6.16) | 33.15 (29.40-37.40) | 14.48-47.48 |
| Guadalajara | 288 | 30.66 (3.79) | 30.66 (28.00-32.80) | 21.9-44.2 | 29.78 (6.73) | 29.78 (25.16-34.59) | 10.86-49.68 |
| Toledo | 204 | 28.65 (2.75) | 28.65 (26.90-30.10) | 20.3-38.9 | 28.10 (4.97) | 28.10 (24.87-31.03) | 16.25-43.09 |
| Badajoz | 165 | 26.01 (2.32) | 26.01 (24.60-27.30) | 20.3-35.8 | 38.24 (4.52) | 38.24 (34.42-41.74) | 24.61-49.71 |
| Cáceres | 223 | 27.21 (2.76) | 27.21 (25.40-28.60) | 21.3-37.7 | 36.24 (5.38) | 36.24 (33.23-40.07) | 17.91-47.35 |
| Barcelona | 311 | 28.17 (3.32) | 28.17 (26.00-30.00) | 20.5-42.3 | 20.09 (5.96) | 20.09 (16.38-23.59) | 6.14-39.63 |
| Girona | 221 | 29.8 (4.10) | 29.8 (26.60-32.30) | 21.6-44.2 | 22.08 (7.19) | 22.08 (16.98-27.02) | 6.31-46.76 |
| Lleida | 231 | 29.27 (3.03) | 29.27 (27.25-30.9) | 22.6-44.2 | 25.43 (6.28) | 25.43 (21.59-28.78) | 6.14-49.91 |
| Tarragona | 184 | 28.8 (2.70) | 28.80 (26.87-30.45) | 20.9-38.3 | 27.71 (6.64) | 27.71 (23.82-32.02) | 12.9-44.14 |
| Alicante | 141 | 30.6 (3.12) | 30.60 (28.50-32.80) | 24.4-38.1 | 33.68 (5.68) | 33.68 (30.72-37.58) | 17.69-45.45 |
| Castellón | 135 | 28.04 (3.10) | 28.04 (25.90-29.85) | 20.6-38.4 | 29.00 (5.92) | 29.00 (24.66-33.41) | 16.53-44.51 |
| Valencia | 266 | 27.72 (2.49) | 27.72 (26.13-29.27) | 21.4-36.8 | 26.30 (6.92) | 26.3 (21.16-30.73) | 12.31-45.63 |
| Balears (Illes) | 6 | 32.03 (2.17) | 32.03 (30.77-33.85) | 28.8-34.3 | 21.90 (2.96) | 21.90 (20.31-22.07) | 19.49-27.61 |
| Menorca | 53 | 30.35 (2.59) | 30.35 (28.70-31.90) | 24.8-40.6 | 23.47 (4.51) | 23.47 (21.52-26.67) | 13.47-31.43 |
| Eivissa y Formentera | 8 | 29.35 (4.10) | 29.35 (27.67-30.57) | 23.1-36.9 | 18.78 (2.93) | 18.78 (16.41-20.96) | 14.17-22.41 |
| Almería | 103 | 29.76 (2.78) | 29.76 (28.05-31.55) | 22.2-36.7 | 37.15 (4.93) | 37.15 (34.55-40.76) | 23.27-46.04 |
| Cádiz | 45 | 28.20 (3.33) | 28.20 (25.50-29.80) | 21.7-37.0 | 36.57 (4.06) | 36.57 (34.38-38.79) | 26.87-44.04 |
| Córdoba | 77 | 26.40 (2.01) | 26.40 (25.10-27.70) | 21.4-32.0 | 35.42 (3.64) | 35.42 (32.92-38.17) | 26.88-43.26 |
| Granada | 174 | 30.03 (3.12) | 30.03 (28.03-31.67) | 23.8-44.2 | 39.07 (5.52) | 39.07 (34.93-43.57) | 25.63-50.64 |
| Huelva | 80 | 27.52 (2.23) | 27.52 (25.88-28.83) | 23.1-34.1 | 34.71 (5.37) | 34.71 (32.37-38.46) | 12.32-48.05 |
| Jaén | 97 | 27.68 (2.09) | 27.68 (26.20-29.10) | 22.5-32.3 | 39.17 (4.25) | 39.17 (36.76-42.58) | 24.66-50.14 |
| Málaga | 103 | 29.87 (3.56) | 29.87 (27.50-32.80) | 23.0-44.2 | 38.09 (4.32) | 38.09 (35.63-41.05) | 21.61-46.42 |
| Sevilla | 106 | 27.47 (2.31) | 27.47 (25.85-28.90) | 22.5-34.3 | 29.90 (6.85) | 29.9 (25.78-34.57) | 11.43-43.52 |
| Murcia | 45 | 28.79 (2.01) | 28.79 (27.50-29.80) | 24.2-33.1 | 35.17 (3.18) | 35.17 (32.73-37.38) | 28.06-41.72 |

N, total number; SD, standard deviation; Q, quartile; Min, minimum; max, maximum.
